# Learning, visualizing and exploring 16S rRNA structure using an attention-based deep neural network

**DOI:** 10.1371/journal.pcbi.1009345

**Published:** 2021-09-22

**Authors:** Zhengqiao Zhao, Stephen Woloszynek, Felix Agbavor, Joshua Chang Mell, Bahrad A. Sokhansanj, Gail L. Rosen

**Affiliations:** 1 Ecological and Evolutionary Signal-Processing and Informatics Laboratory, Department of Electrical and Computer Engineering, College of Engineering, Drexel University, Philadelphia, Pennsylvania, United States of America; 2 Beth Israel Deaconess Medical Center, Boston, Massachusetts, United States of America; 3 Harvard Medical School, Boston, Massachusetts, United States of America; 4 School of Biomedical Engineering, Science and Health Systems, Drexel University, Philadelphia, Pennsylvania, United States of America; 5 College of Medicine, Drexel University, Philadelphia, Pennsylvania, United States of America; University of Washington, UNITED STATES

## Abstract

Recurrent neural networks with memory and attention mechanisms are widely used in natural language processing because they can capture short and long term sequential information for diverse tasks. We propose an integrated deep learning model for microbial DNA sequence data, which exploits convolutional neural networks, recurrent neural networks, and attention mechanisms to predict taxonomic classifications and sample-associated attributes, such as the relationship between the microbiome and host phenotype, on the read/sequence level. In this paper, we develop this novel deep learning approach and evaluate its application to amplicon sequences. We apply our approach to short DNA reads and full sequences of 16S ribosomal RNA (rRNA) marker genes, which identify the heterogeneity of a microbial community sample. We demonstrate that our implementation of a novel attention-based deep network architecture, Read2Pheno, achieves read-level phenotypic prediction. Training Read2Pheno models will encode sequences (reads) into dense, meaningful representations: learned embedded vectors output from the intermediate layer of the network model, which can provide biological insight when visualized. The attention layer of Read2Pheno models can also automatically identify nucleotide regions in reads/sequences which are particularly informative for classification. As such, this novel approach can avoid pre/post-processing and manual interpretation required with conventional approaches to microbiome sequence classification. We further show, as proof-of-concept, that aggregating read-level information can robustly predict microbial community properties, host phenotype, and taxonomic classification, with performance at least comparable to conventional approaches. An implementation of the attention-based deep learning network is available at https://github.com/EESI/sequence_attention (a python package) and https://github.com/EESI/seq2att (a command line tool).

## Author summary

Microbiomes are communities of microscopic organisms found everywhere, including on and in the human body. For example, the gut microbiome plays an important role in digestion, and changes in composition are associated with changes in health or disease, e.g., inflammatory bowel disease (IBD). Today, microbiome composition is often obtained from high-throughput sequencing, which generates many short DNA reads from multiple organisms in a sample. In this paper, we present a novel deep learning framework, Read2Pheno, to predict phenotype from all the reads in a set of biological samples. An attention mechanism allows visualization of specific subregions (sets of bases) which are important in classifying the reads according to phenotype or taxon labels. We evaluate the framework on sequencing data for 16S rRNA genes, genetic markers used to identify microbial taxonomy. We show that Read2Pheno performs comparably as conventional methods on three distinct data sets from the American Gut Project, IBD patients and controls, and a comprehensive taxonomic database. Moreover, Read2Pheno results can be readily interpreted—e.g., to identify regions of the 16S rRNA gene to target for PCR diagnostics—without additional pre/post-processing steps that can introduce complexity and error.

This is a *PLOS Computational Biology* Methods paper.

## Introduction

Advances in DNA sequencing are rapidly producing complex microbiome data sets in fields ranging from human health to environmental studies [1]. Large-scale microbial projects provide rich information, enabling prediction of sample-level traits (i.e., phenotypes), aiding biological discovery, and supporting medical diagnosis. A typical microbiome study may contain hundreds to thousands of samples. Each sample, in turn, contains thousands of reads depending on the sequencing depth. These reads are fragments of DNA/RNA material extracted from microbes residing in the environment where the sample was collected. For example, an environmental sample can be sequenced via 16S ribosomal RNA amplicon technology, to provide a comprehensive taxonomic survey of an environment’s or subject’s microbial community [2, 3].

A major focus of microbiome research has been, and continues to be, the use of 16S rRNA amplicon sequencing surveys to determine “Who is there?” in a host or environmental sample. The answer to “Who is there?” may, in turn, be used to predict host phenotype for clinical diagnoses or infer taxa-phenotype association for basic biology research [4–8]. In the context of our work, we define “phenoytpe” as an overall trait at the environmental level or habitat that the microbiome sample is isolated from [9, 10], thereby incorporating the emergent function of the microbiome (a.k.a. microbiome phenotypes) [11–16]. For example, the expansive definition of “phenotype” in the microbiome context can include the preference of a certain microbial community for a particular environmental niche or body site [17]. Thus, the microbiome may be shaped by the environment.

While shotgun metagenomic sequencing technology may be used instead [3, 18, 19], many use cases depend on 16S rRNA amplicon sequencing as an affordable, rapid, and readily field-deployable solution to find out “Who is there?”. However, *phenotype prediction* from rRNA sequence is a major challenge. Ribosomal sequence does not itself contain functional information, unlike, e.g., more costly and complex metagenomic shotgun sequencing data [7, 20]). Building machine learning phenotype classifiers usually starts with constructing a microbial abundance table, such as an Operational Taxonomic Unit (OTU) table, an Amplicon Sequence Variant (ASV) table, or a *k*-mer frequency table (i.e., table of the frequencies of k-length nucleotide strings within the collection of reads in a sample) [6, 7]. Researchers then train a classifier to distinguish phenotypes by learning from the taxon abundance of sequenced samples in a training data set. For example, a classifier may be constructed to identify a sample as being from a patient’s gut who was diagnosed with a disease.

By analyzing the OTU/ASV abundance table, therefore, researchers can discover underlying associations between certain taxa or groups of taxa and phenotype. For example, in Gevers *et al.* [5], samples were collected from a) patients with Crohn’s disease and b) control groups, and some taxa were found to only be abundant in the disease group, and some were eliminated in disease group. In another study, 16S rRNA sequences were transformed to OTU tables to evaluate 18 classification methods and 5 feature selection methods, and feature selection was shown to often improve classification performance [21]. Another classifier method based on RoDEO (Robust Differential Expression Operator) normalization was shown to sometimes perform better when a small subset of OTUs were used [6].

The construction of OTU/ASV tables, however, often involves denoising, sequence alignment, and taxonomic classification, and thus can lead to information loss from the true information contained in the raw nucleotide reads. And, as shown above, it can require additional steps of processing, for example feature selection or OTU table reduction. By grouping sequences to limited taxonomic labels, it becomes difficult to quantify the genotype-to-phenotype relationship. Of particular concern is the omission of nucleotide structural information from OTU mapping, where the 97% identity threshold conventionally used for OTU mapping smooths over valuable nucleotide variation. This is better addressed through the more exact ASV identification—but rarely is the nucleotide level information examined past the mapping step. Alternatively, a *k*-mer representation of amplicon sequences has been proposed to predict phenotype, which is shown can outperform traditional OTU representation [7]. Since a *k*-mer-based method is alignment free and reference free, it would cost less computationally than OTU-based methods if relative small *k*-mer size is used (e.g., *k*-mer values of 3 ≤ *k* ≤ 8 are typically used in MicroPheno [7]). Because *k*-mer representations cut reads into smaller pieces, methods based on *k*-mers will lose sequential information. As such, *k*-mer analysis is subject to the length of the *k*-mers and does not preserve the nucleotide context/sequential-order. Some local nucleotide variation may be able to be identified; however, the long-range nucleotide sequential information is completely lost. In sum, currently available methods are unable to easily and robustly connect nucleotide changes on the read level back to the phenotype prediction and thereby reveal which nucleotide features are specifically relevant to the classification.

### Deep neural networks and their application in bioinformatics

Recent advances in supervised deep learning are further able to leverage a huge volume of different kinds of data. Convolutional neural networks (CNNs), which may be interpreted by saliency maps [22], have been vital to image recognition. Model interpretability has been a research direction of particular interest in the deep learning field [23–25]. Deep learning has been applied to bioinformatics too [26]. Deep learning approaches have been shown to be able to learn hierarchical representations of metagenomic data that standard classification methods do not allow [27]. Both CNNs and RNNs have been applied to areas such as transcription factor binding site classification [28, 29], SNP calling [30, 31], microbial taxonomic classification [32] and DNA sequence function prediction and gene inference [33, 34]. Other work has used deep learning approaches to predict environments and host phenotype using *k*-mer-based representation of shallow subsamples [7]. Lo *et al.* proposed deep learning approaches to learn microbial count data (e.g., OTU table) for host phenotype prediction [35], and another approach formats microbial count data as an “image” to be processed by a CNN model [36]. CNN models were used to learn phylogenetic structure of a metagenomic sample to predict the host phenotype [37]. A 2D matrix is used to represent the phylogenetic tree of microbial taxa (with relative abundance) in a sample, and a CNN model is designed to learn from such data. Woloszynek *et al.* proposed an unsupervised method to embed 16S rRNA sequences to meaningful numerical vectors to facilitate the down-stream analysis and visualization [38]. Many models rely on extracting “features” (for instance, taxonomic composition or functional profiles) from the sequence data [39].

In addition to making predictions, machine learning models can reveal knowledge about domain relationships contained in data, often referred to as interpretations [40]. In the context of sequence classification tasks, i.e., microbial survey data based phenotype prediction, once a predictive model is built, the researchers can further identify sequence features relevant to classifications, i.e., occurring taxa and gnomic content related to a certain disease. There are substantial research attempts to identify label associated genetic content. A complementary approach is *supervised* computational method, as a means of associating genetic content with known labels, i.e., taxa. “Oligotyping” has been proposed as a way to identify subtypes of 16S rRNA sequence variation, based on distinguishing sequence variants by subsets of several nucleotides within the sequence, i.e., oligomers. Specifically, *Oligotyping* is a supervised computational method that identifies those nucleotide positions that represent information-rich variation [41]. *Oligotyping* requires information about the taxonomic classification of the sequence via OTU clustering or supervised methods. Then, the method is applied to taxonomical/OTU groups of interest. *Oligotyping* can be an efficient way to identify informative nucleotides and discriminate between closely related but distinct taxa [41]. However, preprocessing steps are still needed (e.g., OTU clustering or multiple sequence alignment) to find closely related sequences.

Another proposed method, “PhenotypeSeeker” [42], is a statistics-based framework to find genotype-phenotype associations by identifying predictive *k*-mers by a regression model and quantifying their relative importance. This was designed, however, to learn a closely related group of bacterial isolates and their associated phenotypes. Furthermore, “PhenotypeSeeker’’ and other *k*-mer frequency tables based methods cannot capture the sequential order information of *k*-mer, and thus they fail to provide sequence-level interpretability. Visualization methods are developed for DNA/RNA binding sites prediction models as mentioned in Section Deep neural networks and their application in bioinformatics [28, 29, 43, 44] to reveal predictive genomic content. Alipanahi *et al.* propose to interpret the model and visualize informative single nucleotide polymorphisms (SNPs) by manually altering nucleotides in the input reads and comparing the resulting new prediction with the original prediction of the unaltered input [43]. In *Deep Motif*, the authors use Saliency Maps [22, 25] to interpret the model and visualize informative genomic content [28].

### Towards better interpretability: Attention mechanisms

Attention mechanisms have become more widely applied in the natural language processing (NLP) and image recognition fields to improve the interpretability of deep learning models [45–48]. For example, it has been shown that an attention-based Bi-LSTM (Bi-directional long short term memory) RNN model can successfully capture the most important semantic information in a sentence and outperform most existing competing approaches [47]. A hierarchical attention network can also improve document level classification [46] by selecting qualitatively informative words and sentences. Informative content may be visualized by looking at the output of the attention layers of the network model. The use of deep learning with attention mechanisms has also been suggested for the field of bioinformatics. Deming *et al.* [29] proposed a method for simultaneously learning general genomic patterns and identifies the sequence motifs that contribute most to predicting functional genomic outcomes, e.g., transcription factor binding site (TFBS) classification and lineage-specific gene expression prediction. While they found a marked gain in performance over previous architectures, their model was not used for phenotype prediction.

In this paper, we exploit CNNs, RNNs, and attention mechanisms for phenotype/taxonomic prediction and propose a Read2Pheno classifier to predict phenotype from 16S rRNA reads and, thereby, explore and visualize informative nucleotide structure and taxa. This method can be considered as a supervised read-level embedding method compared with our previous work in word2vec embedding methods for 16S rRNA reads [38]. Although the model focuses on read-level resolution, the sample-to-phenotype prediction can still be inferred by a sample-level predictor which aggregates the abstraction of all reads from the Read2Pheno model. A python implementation of the proposed model is available at https://github.com/EESI/sequence_attention. A command line tool of the proposed model is avaiable at https://github.com/EESI/seq2att. We show that the model trained with read-level information can achieve similar sample-to-phenotype predictions compared with conventional methods. Our proof-of-concept results demonstrate the potential of our proposed read-level training procedure to provide the basis for more accurate and comprehensive sample-level classification, as compared to OTU tables, and substantially more interpretable results than *k*-mer-based methods. We further provide a visualization of the embedded vectors, which is a representation of the information that the network is learning. We use attention weights to identify and visualize the nucleotides associated with phenotype and/or taxonomy, and compare the highlighted informative regions against a base-line entropy method and *Oligotyping* [41].

We show the efficacy of our model with the American Gut microbiome data set [49] (http://americangut.org/), Gevers *et al.*’s Crohn’s disease data set [5] and SILVA 16S rRNA data set [50, 51] and explore interesting visualizations and features generated by the model. The experimental results show that the performance of our model is comparable to current methods and our models can provide further interpretation and visualization.

## Materials and methods

**Fig 1:**
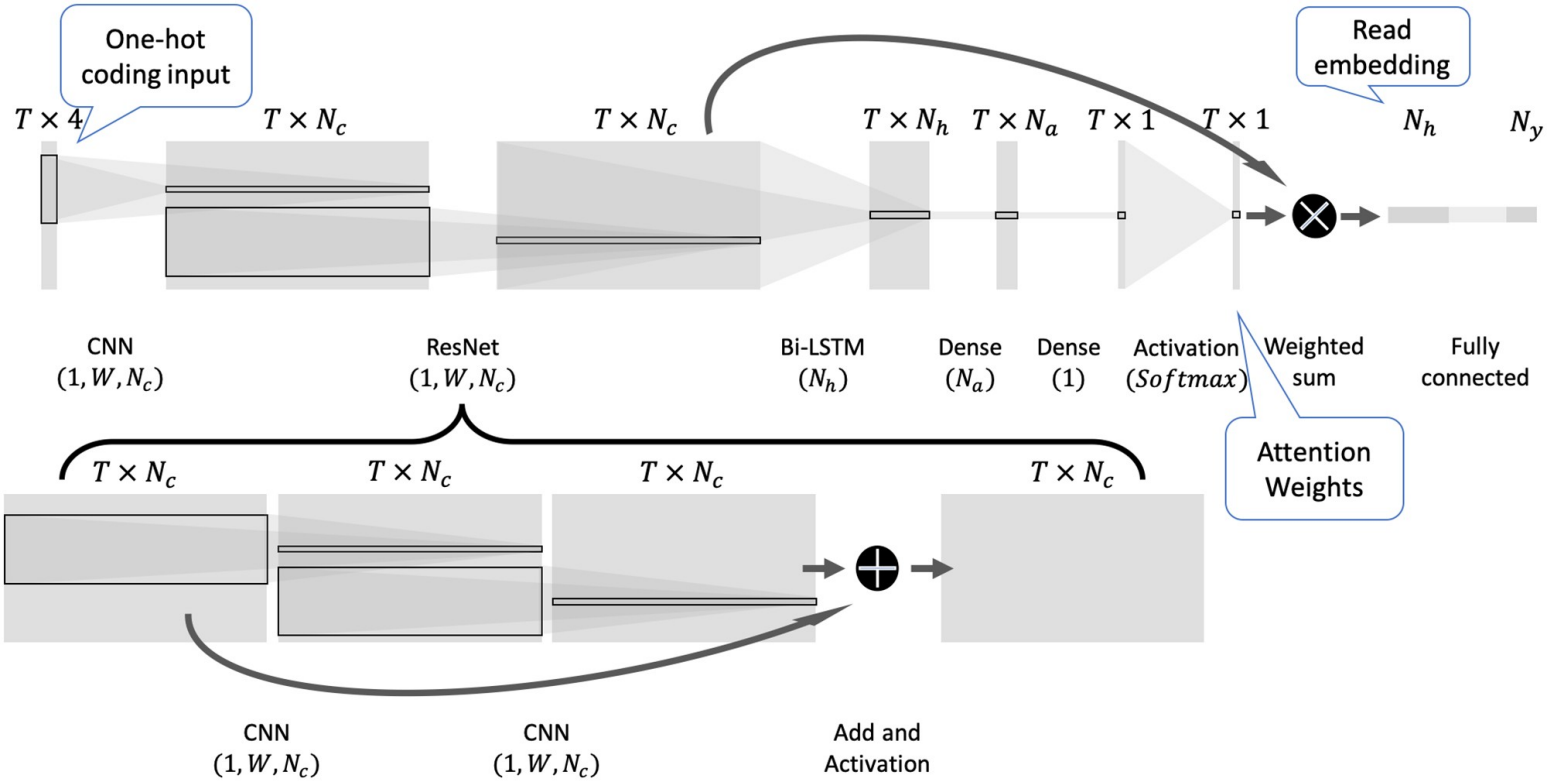
Read2Pheno classifier architecture: The input is a one-hot coded 16S rRNA sequence with length *T*. The input is fed to a few 1-dimensional convolutional blocks with window size of *W* and the number of output channels of *N*_*c*_. The resultant output is a *T* × *N*_*c*_ dimensional matrix which is then fed to a Bidirectional LSTM layer with the number of hidden nodes of *N*_*h*_. *N*_*a*_ is the number of hidden nodes used to compute attention weights and *N*_*y*_ is the total number of phenotypes (number of classes) to be predicted. There are two informative intermediate layer outputs (attention weights and read embedding vectors) which are labeled by speech balloons. They are used in the analysis described in this paper.

Our proposed model consists of two parts: 1) the Read2Pheno read-level classifier, which is the focus of our paper, and 2) several sample-level predictors based on the Read2Pheno model, primarily used here to demonstrate the evaluation of the Read2Pheno model. We first train a read-level classifier using an attention-based deep neural network to predict DNA/RNA reads to the sample-level labels the reads associated with. For example, if the samples are labeled with collected body sites, the model will be trained to learn the original body site from which the samples’ reads were collected. Then, a sample-level prediction can be made by three different ways: 1) tally a majority vote of all the read prediction scores in the sample of interest to obtain a final prediction; 2) use the output of the intermediate layer to obtain a read embedding (see Fig 1 for details) and average read embeddings from a sample to gain an overall sample-level embedding that a classifier can train on to predict a sample-level label; 3) apply clustering on read embeddings of training data and assign reads per sample to those clusters to form a “Pseudo OTU” table [38]. Then, a sample-level classifier can be trained for phenotype prediction, which allows validation of the read-level model, i.e., by showing that it can produce accurate and relevant phenotype predictions.

### Read2Pheno classifier

The Read2Pheno classifier is a hybrid convolutional and recurrent deep neural network with attention. Fig 1 shows a diagram of the classifier. Sequencing data are one-hot coded according to the map shown in S1 Appendix. Then the array representation of a read is fed into several initial layers of convolutional blocks (inspired by the scheme in [29]). The result is an embedding of the read, a *T* × *N*_*c*_ dimensional matrix, by learning local *k*-mer patterns, where *N*_*c*_ is the number of output channels in convolutional blocks and *T* is the length of input DNA reads. A Bi-directional Long Short Term Memory (Bi-LSTM) model is then applied to the data to learn the longitude dependency of the output of the convolutional layers. The returned sequence is then processed and normalized by an attention layer to get an attention vector using the soft attention mechanism, as described in [47, 52]. The output of Bi-LSTM layer in our model is a *T* × *N*_*h*_ dimensional matrix where *N*_*h*_ is the number of hidden nodes in Bi-LSTM layer and *T* is the length of input DNA reads. Each base position (time-step) in the input corresponds to an *N*_*h*_ dimensional vector (hidden states at this position). The dense attention layer applies to the hidden states of every base position (time-step). The dense layer thereby learns the importance of hidden states at each position and return a small value if the hidden states of this position do not make an important contribution to the model’s final prediction, and, conversely, a large value if the model relies on the hidden states at this position in making the final prediction. The output of the dense layer is a vector of length *T*. Then, the output is normalized by a softmax function to produce the attention vector [52]. The output of this layer naturally indicates the regions in the sequence that the model pays attention to. While the attention weights are not learned from specific nucleotides but from high level features from 9-mers and their sequential information, as shown in Fig 1, the attention interpretation may be considered to be an approximation of the informative nucleotides of the 16S rRNA gene. The final embedding of the read is a weighted sum of all the embeddings across the sequence, where the weights are the elements of the attention vector. The goal of this layer is to suppress the regions that are less relevant to prediction and focus on informative regions. Finally, a dense layer with softmax activation function is applied to the read embedding vector to classify it into one of *N*_*y*_ labels. The hyperparameter selection process is described in Section Model selection and hyperparameter search.

### Sample-level predictor

In this paper, we perform sample-level prediction in three different ways. The simplest of the three is majority vote. The sample-level predictor counts all the votes, i.e., the resulting Read2Pheno classifications, from all the reads in a query sample and labels the sample with the overall majority vote. The majority vote is a baseline method intended to illustrate that the Read2Pheno model is learning the sample-associated phenotypic labels for each read. We compare the majority vote baseline to proposed embedding-based approaches further described below.

The intermediate layer of our model provides a concise numerical representation of the input reads, which we can exploit in sample-level prediction. We propose to use two embedding based approaches: sample-level embedding method and “Pseudo OTU” method [38]. The sample-level embedding method forms a sample-level vector representation by averaging all read-level embeddings in a query sample. Then, a classifier, such as Random Forest, can be trained to learn the sample-phenotype association. For the Pseudo OTU method, as described by Woloszynek *et al.* [38], firstly read-level embedding vectors are clustered via an unsupervised algorithm such as k-means to form *k* clusters that are Pseudo OTUs (groupings of related reads). Then, we can assign each query sample’s reads to those Pseudo OTUs based on distance. A classifier, such as Random Forest, can then be trained to make sample-level predictions on a Pseudo OTU table made up of the Pseudo OTU abundance, as defined above, in all samples. Both embedding-based methods learn the sample phenotype by training on each individual read (“read-level”) and on all reads (“sample-level”) rather than read-level-only learning, as for baseline majority vote.

#### Majority vote

The Read2Pheno classifier produces a vector of likelihood scores which, given a read, sum to one across all phenotype classes. To get the sample-level prediction, all reads from a sample of interest are classified by Read2Pheno model, and the resultant scores are then aggregated by the sample-level predictor. Using body site prediction as an example, there are 5 different body site classes: feces, tongue, skin of hand, skin of head and nostril. We show the diagram of our sample-level predictor in S2 Appendix. Given a sample of interest, the reads associated with this sample are first predicted by Read2Pheno classifier. Some species can be found in multiple body sites. Therefore, performing a hard call on which body sites a read originates from can be misleading. To alleviate this problem, if needed, the sample-level predictor contains a read caller function that can assign one read to multiple body sites by applying a threshold to the output of Read2Pheno for the read. In our implementation, if the likelihood score of the read from a body site is greater than chance ($\frac{1}{N}$, where *N* is the number of body sites in the training data), the vote count of that particular body site will increment by 1 (see the “Read Abundance” block in S2 Appendix). For example, suppose there are three target body sites: skin (i.e., dermal samples), gut (i.e., fecal samples), and tongue (i.e., oral samples). If a read were predicted to be from gut, skin and oral samples with scores of 0.51, 0.43 and 0.06 respectively, both the vote counts of feces class and skin class would increment by 1 (since the likelihood of these two body sites are greater than $\frac{1}{3}$). Finally, once all reads have been counted, the argmax of vote count vector is taken to predict the sample-level body site.

#### Embedding-based method

The attention layer of the Read2Pheno classifier produces an *N*_*h*_-dimensional embedded vector (see Fig 1), which is a meaningful numerical representation of each 16S rRNA read. For sample-level classification, we first use the trained Read2Pheno model to encode all reads per sample into the *N*_*h*_-dimensional vectors. We describe two methods to produce sample-level features using the read embeddings: the averaged sample-level embedding and Pseudo OTU table.

**Averaged sample-level embedding:** To obtain a sample-level embedding, we first use the trained Read2Pheno model to encode all reads per sample into the *N*_*h*_-dimensional vectors. Then, we average the read vectors to form a sample-level embedding.

**Pseudo OTU table:** Instead of taking the average of the trained read embeddings, we use a *k-means* algorithm with the default Euclidean distance metric to cluster the read embeddings of training data into 1000 clusters [38]. Then, all reads in each query sample can be assigned to those clusters. Effectively, the clusters represent related sequences, which are called Pseudo OTUs. We compute the number of reads assigned to each Pseudo OTU for each sample to create a Pseudo OTU table: a matrix of Pseudo OTUs versus samples.

We can then train a classifier (e.g. Random Forest) on the sample-level features to predict phenotype. We show the training and testing process of averaged sample-level embedding and Pseudo OTU table based sample-level prediction in S3 and S4 Appendices respectively.

#### Traditional sample-level classifiers

We also train and evaluate Random Forest classifiers using 1) *k*-mer frequency tables, 2) OTU abundance tables and 3) ASV tables generated by Dada2 [53] to show that our proposed model can extract meaningful features on the read level, which can then be used to achieve comparable performance at the sample level. In our experiments, all the tables are first normalized by the row sum to create relative abundance table which are used as the input to the random forest classifiers. We use the scikit-learn implementation of Random Forest classifier with default parameters (number of estimators = 100).

#### Pretrained word2vec embedding based sample-level classifiers

Woloszynek *et al.* published a pretrained Skip-Gram word2vec model that was trained on 2,262,986 full-length 16S rRNA amplicon sequences from the GreenGenes reference database for 10-mers [38]. Since each k-mer is mapped to a numerical vector, the read-level embedding is obtained by averaging all numerical vectors that corresponds to 10-mers in a read. The sample-level embeddings can then be obtained using the same methods described in Embedding-based method. Downstream Random Forest classifiers trained on the embeddings may serve as additional baselines. The pretrained word2vec method can in general be classified as a *k*-mer based methods as well, since the word2vec model is trained to predict neighbor *k*-mers given a *k*-mer input. However, this method can leverage the information from millions of 16S rRNA reference sequences. As such, the pretrained word2vec method provides a good baseline to show how well embedded vectors obtained using this paper’s attention-based method can encode information.

### Data preparation for model evaluation

#### American Gut Project (AGP) data set

The AGP data set used for model evaluation in this paper is a subset of data from the American Gut Project [49]. As of May 2017, American Gut project reported that AGP included microbial sequence data from in total 15,096 samples from 11,336 human participants (subjects) and that number continues to grow as the project is ongoing [49]. We focus on samples from five major body sites (*N*_*y*_ = 5): feces, tongue, skin of hand, skin of head and nostril. As mentioned in American Gut Project’s documentation, some bloomed organisms were contained in samples analyzed early in the American Gut Project because of increased shipping time and delay between when samples were collected and when they were put on ice. As a result, bloom sequences have been removed in preprocessing process by American Gut Project. In this study, we use the latest filtered sequences and OTU table deposited in ftp://ftp.microbio.me/AmericanGut/latest as of 2018/12. All reads have been trimmed to 100 base pairs by American Gut Project, so that *T* = 100 in Fig 1.

#### Gevers data set

The Gevers data set used for model evaluation in this paper is a subset of an inflammatory bowel disease (IBD) data set [5] (NCBI SRA index: PRJNA237362 in NBCI). Sample metadata label them as being IBD or Non-IBD (*N*_*y*_ = 2). Here, we refer to IBD samples as “CD” (Crohn’s Disease), and the Non-IBD ones as “Not IBD” (disease-negative). We merge paired reads using QIIME [54] and trim them to 160 base pairs (i.e., with the first 10 removed, the following 160 base pairs kept and the rest discarded), so that *T* = 160 in Fig 1. To confirm that the Read2Pheno model would be robust to a longer read, we also evaluated reads trimmed to 250 bp (see S16 Appendix).

#### Experimental setup for American Gut Project data set and Gevers data set

First, we filter out samples with less than 10,000 reads. Then, we randomly select 161 samples from American Gut Project data set and 221 samples for Gevers data set per class as our experimental data set to balance the class distribution (resulting in total 805 samples in AGP experimental data set and 442 samples in the Gevers experimental data set). The number of samples are selected based on the least number of sample per class after filtering for each data set. Next, we randomly select a certain number of samples per class for training and leave out the rest for testing. For the AGP data set, 10, 80 and 150 samples per class are randomly selected for training (resulting in 50, 400 and 750 samples total respectively). For the Gevers data set, 20, 80 and 200 samples per class are randomly selected as training data (resulting in 40, 160 and 400 samples total respectively).

For the AGP data set-based experiment used for attention interpretation, we randomly select 10 samples per class for training, resulting, in total, 50 samples and 1,503,639 reads for training. The rest of the samples form the testing data set. Metadata for this experimental data set are available in S5 Appendix, where we provide additional information about the hosts such as the race, sex and health status. We randomly select 10 samples per class as the candidate visualization set. For the Gevers data set-based experiment used for attention interpretation, we select 40 samples (20 from the IBD class and 20 from non-IBD) by random and collect 1,678,464 reads for training (around 42,000 reads per sample). The remaining samples (442—#_of_training) are used for testing. We again randomly select 10 samples per class from the testing data set as the candidate visualization set. After we select the candidate visualization set for both attention interpretation experiments, we use the QIIME [54] implementation of the Ribosomal Database Project (RDP) [55] taxonomic classification with GreenGenes v13.8 database to assign the genus-level labels to reads in the candidate visualization set. Then, reads with less than a 80% RDP confidence score on genus level are removed from the visualization set.

Finally, in order to efficiently extract intermediate layer outputs and generate visualizations, an arbitrary subset, 100,000 reads from the qualified visualization set, are randomly sampled for the final visualization and interpretation. All reads in the final visualization set have a genus-level label and phenotype (i.e., body site or disease diagnosis) label. For the AGP visualization set, we further merge the skin-associated label, namely, skin of head, skin of hand and nostril into one single skin class to simplify the visualization. As a result, the visualization set reflects 3 body site classifications instead of 5. We use the experimental setup for the AGP data set as an example to show the overall training and testing experiment in S6 Appendix.

#### SILVA data set and experimental setup

The SILVA 16S taxonomic QIIME-compatible data set is used to construct our experimental data set [50, 51]. There are 369,953 sequences total in the original data set. Among those sequences, there are 268,225 which have a genus-level label, and those sequences come from 6,618 genera. We select the genera that have over 100 representative sequences and collected all sequences from these genera to form our experimental data set. We thereby include in total 204,789 sequences from *N*_*y*_ = 495 genera in our experimental data set where *N*_*y*_ is the number of target classes in taxonomic prediction. Our data set covers around 76.35% sequences and 7.48% of the genera in original data set. Sequences are first one-hot coded according to the map shown in S1 Appendix. Then, we right-pad the sequences with zero vectors to the nearest hundred and group sequences based on the padded length (resulting 11 groups that have 100bp increment size in the range of *T* = 900 → 1900). For example, a sequence of length 1001 will be padded with 99 zero vectors to a total length of 1100bp. Then, those sequences are stored in the same matrix per length group. In this way, model can be trained by sequences with similar length at a time to improve the training efficiency. We then randomly split the data set into 80% sequences as training and 20% as testing.

### The Read2Pheno training process

We train the Read2Pheno model with reads from training data set, preprocessed and selected as described above, labeling reads by sample phenotype. Since the Read2Pheno model should be trained and optimized for read-level prediction, sample-level predictors are trained separately after the Read2Pheno model training is completed. For each testing sample, all reads are classified by the Read2Pheno model. We then aggregate the read-level information encoded by the Read2Pheno model using methods described in Section Sample-level predictor to make sample-level predictions. We show a schematic of the training process in S7 Appendix.

We randomly sample an equal number of samples from each class to form the training set. Then, we label all reads associated with those samples by their sample-level label and shuffled. All reads are one-hot coded according to the coding map in S1 Appendix. Then, the data are fed to the Read2Pheno model for training. The reason we train our neural network on the read level instead of sample level is two-fold: 1) our read-level model can highlight informative regions in each input sequence; 2) there are relative less number of examples to train a complex neural network model on the sample level than the read level. As discussed in Section Model selection and hyperparameter search, we further show that the read-level model trained with a dozen of samples performs comparably to read-level model trained with hundreds of samples.

Our deep learning model is implemented in Keras (version 2.2.2) with Tensorflow (version 1.9.0) backend. If the number of classes is greater than 2, then categorical cross-entropy can be used as the loss function. Otherwise, binary cross-entropy is the recommended loss function. Adam optimization with default setting and a learning rate of 0.001 is used to train the model. The model was trained and evaluated on the Extreme Science and Engineering Discovery Environment (XSEDE) [56] for 10 epochs. We also made the proposed Read2Pheno model available in Github at https://github.com/EESI/sequence_attention (a python package) and https://github.com/EESI/seq2att (a command line tool).

### Model interpretation and read visualization

The Read2Pheno model has an LSTM layer; consequently, sequential information are encoded and circulated in hidden states. The intermediate output, labeled as “Read embedding” in Fig 1 is an *N*_*h*_-dimensional vector. This read embedding vector is an average of hidden states across all bases weighted by the attention weights, labeled as “Attention Weights” in Fig 1. The *N*_*h*_-dimensional embedding vector can be considered as a numerical representation of the input DNA/RNA read. Therefore, similar reads from the same taxonomic group should be embedded to vectors that are close to each other in *N*_*h*_-dimensional space, whereas differing reads should be embedded far away from each other. This type of relationship may be shown by plotting the *N*_*h*_-dimensional vector representations of the reads in a 2-dimensional space. Accordingly, we use Principal Component Analysis (PCA) to reduce the dimensionality of all reads in visualization set to 2-dimension by projecting them onto the top 2 principal components that explain the most variation. In this study, we use the QIIME [54] implementation of the RDP [55] taxonomic classification method to predict the genus level label for our visualization reads.

Inspired by “WebLogo” [57, 58], we also use a “sequence logo” to visualize significant features contained by the sequence. The reads from same genus are similar to each other, with mutations at certain positions. We thus group the visualization reads from the same genus together for further exploratory visualization.

We calculate the overall Shannon Entropy of a group of reads (reads from a genus) by:

$$\begin{array}{c} H\left(l\right)=-\Sigma_{b}p\left(b,l\right)\cdot log_{2}\left(p\left(b,l\right)\right) \end{array}$$ (1)

where *b* is the nucleotide base, *b* ∈ {*A*, *T*, *G*, *C*}, *l* is the position of the sequence, *l* ∈ (0,length(seq)]. *p*(*b*, *l*) can be estimated by *f*(*b*, *l*), the normalized nucleotide frequency of base *b* at position *l*. For our phenotype prediction models, the sequence logo for a given phenotype can be calculated by Eq 2 where *c* is the phenotype label and *f*_*c*_(*b*, *l*) is the normalized nucleotide frequency of base *b* at position *l* among reads from phenotype *c*.

$$\begin{array}{c} S_{c}\left(b,l\right)=f_{c}\left(b,l\right)\cdot H\left(l\right) \end{array}$$ (2)

Positions with high nucleotide variance will have high entropy and therefore the sequence logo is a good measure of “importance” (variation) of nucleotides, which we use as our baseline importance measure.

As presented in Fig 1, a dense attention layer learns to predict the importance of bases by hidden states output from Bi-LSTM layer. The output of the attention layer, “Attention Weights”, is a vector of input read length, *T*, wherein each value represents the importance of the hidden state corresponding to said position. This vector will indicate what region of the input sequence the model has been found to be most informative. Therefore, we use the attention weights for the input reads as the model’s predicted importance measure. Among reads from the same genus, the attention weights for reads from the same phenotype are averaged. The mean attention weight vector highlights the informative sequence regions for a phenotype for this genus. The attention measure is thus defined by Eq 3.

$$\begin{array}{c} A\left(c,l\right)=att_{mean}\left(c,l\right) \end{array}$$ (3)

where *att*_*mean*_(*c*, *l*) is the mean attention weight of reads from a phenotype *c* at position *l*. Note that the same read can be extracted from samples with different phenotype labels, but the model is forced to classify the read to one of the phenotype labels. In order to understand which regions are considered “important” by the model when making a particular prediction, we use the predicted class label as the phenotype label when calculating the mean attention weights in Eq 3 for both AGP and Gevers dataset. For genus classification model trained on SILVA data set, we use the true genus label as the class label to group sequences to compute the mean attention weights because one sequence is associate with only one genus label.

## Results

As described in detail below, we analyzed three distinct 16S rRNA amplicon sequence data sets: 1) data provided by the American Gut Project (AGP), in which samples are labeled by body site origin and thereby reflect microbiome phenotype (i.e., properties of a microbial community); 2) data published by Gevers *et al.* (Gevers), which is labeled by disease diagnosis, i.e., host phenotype; and 3) the SILVA rRNA database, a large corpus of comprehensive and quality checked 16S rRNA sequences with taxonomic labels. Our goals for each data set were to evaluate 1) the performance of attention-based deep learning models at predicting phenotype and taxonomy as compared to existing baseline methods, and 2) interpretability gains afforded by intermediate layer outputs of attention-based deep neural networks through visualizing the ordination of sequence embedding vectors and informative regions of sequences highlighted by attention weights.

### Microbiome phenotype (body site) prediction based on American Gut Project (AGP) data

We evaluated our proposed Read2Pheno attention model on a subset of American Gut Project (AGP) data, which contains sequencing data from the largest crowd-sourced citizen science project to date [49]. The subset contains 805 samples obtained from five body sites: feces, tongue, skin of hand, skin of head, and nostril.

#### Model selection and hyperparameter search

To evaluate model architectures and fine tune hyperparameters, we use the training data for 50 samples to perform a 5-fold cross validation to fine tune the hyperparameters of our model. The reads from 50 randomly selected samples are split into 5 folds. The model is trained with 4 folds and then validated by the remaining fold. The validation accuracy is used to find the best set of parameter and model architecture. The hyperparameter search space can be found in S8 Appendix. The 5-fold cross validation yielded *N*_*c*_ = 256 filters in CNN layers, *N*_*h*_ = 64 units in LSTM layer, dropout rate of 0, and learning rate of 0.001 as the hyperparameters as producing the best read-level classification accuracy on the training data set, and we incorporated them in our model. We also performed the same hyperparameter sweep on other models with related architectures: the Bi-LSTM model, Attention-based Bi-LSTM model, CNN model, Attention-based CNN model, CNN-Bi-LSTM model and Attention-based CNN-Bi-LSTM model. We use the same architecture for CNN and Bi-LSTM layers in the models described in Fig 1. Table 1 shows the best set of hyperparameters for all 6 models. As Table 1 shows, classifiers constructed with only a Bi-LSTM layer or a CNN layer have suboptimal accuracy compared to more complex models. With the help of an attention mechanism, the CNN model achieves better accuracy, but the Bi-LSTM model does not benefit from the attention layer. The classifier which combines CNN layers, a Bi-LSTM layer and an attention layer results in the best accuracy classification following 5-fold cross-validation. Although the model without an attention layer achieves similar accuracy, the interpretability of the attention-based model is superior, as shown in the results below for sample-level prediction. S9 Appendix shows the training and validation loss of the model with the best hyperparameters.

**Table 1:** Comparison of optimal hyperparameters and maximum validation accuracy for different model designs. The optimal hyperparameters and validation accuracy when using those hyperparameters were calculated, based on 5-fold cross validation, for alternative classifier models, i.e., Bi-LSTM alone, Bi-LSTM with an attention (ATT) layer, CNN alone, etc. Hyperparameter search space is described in S8 Appendix. The #Param column shows the number of parameters of the models. The CNN column shows the optimal number of convolutional filters, *N*_*c*_. The RNN column shows the optimal number of hidden nodes in Bi-LSTM, *N*_*h*_. DP refers to the dropout rate (probability of training to a particular hidden node in the layer) and LR is the learning rate (amount weights are updated in each step) used in Adam optimizer. From this table, we observe only small differences in validation accuracy for the different combinations of parameters in S8 Appendix.

Model	#Param	CNN (*N*_*c*_)	RNN (*N*_*h*_)	DP	LR	Acc (± Std)
Bi-LSTM	136,837	-	128	0.25	0.005	0.734 (±0.002)
Bi-LSTM+ATT	138,918	-	128	0.25	0.005	0.732 (±0.003)
CNN	513,541	128	-	0.25	0.001	0.738 (±0.001)
CNN+ATT	1,789,222	256	-	0.25	0.001	0.740 (±0.001)
CNN+Bi-LSTM	2,178,693	256	128	0.25	0.001	0.742 (±0.001)
CNN+Bi-LSTM+ATT	1,949,542	256	64	0	0.001	0.742 (±0.001)

To evaluate the influence of the training size for Read2Pheno classifier training, we designed an independent experiment: we first hold out 55 samples for testing, then we train the Read2Pheno model with reads from 5, 25, 50, 100, 500 and 750 samples from the rest of samples and evaluate the sample-level performance of those models by the 55 samples in the held-out testing set. (Here, we use the sample-level embedding method for sample prediction). To determine how much sample-level prediction quality depends on the size of the Read2Pheno training set as compared to the size of the downstream sample-level training (i.e. Random Forest) set, we measure test set prediction accuracy where sample-level Random Forest is trained 1) on the same samples used to train the Read2Pheno classifier, and 2) on all 750 samples irrespective of the number of samples used to train Read2Pheno. The results are plotted in S10 Appendix. We found that, as more samples are used to train the sample-level (Random Forest) classifier, performance improves. However, increasing the number of samples used to train the Read2Pheno classifier does not generally improve the accuracy of sample-level classification. This suggests that the Read2Pheno classifier can learn a meaningful embedding with only a small number of samples. Indeed, there are usually a great number of reads in a few samples. For example, in the AGP data set, there are over 1 million reads in 50 training samples. Therefore, for further downstream analysis, including visualizations of the embedding and attention weights, we use a Read2Pheno model trained with 50 samples.

#### Sample-level attribute prediction

Sample-level attributes, here the “phenotype” of the host-microbiome interaction, i.e., body site of the microbiome, on the read/sequence level are predicted by sample-level predictors as described in the Methods section (see Sample-level predictor). Table 2 compares the accuracy of our deep learning approaches (a Read2Pheno model trained for 10 epochs with various sample-level classification strategies) against Random Forest baseline approaches, which are trained on four different types of features: Random Forest trained on (1) *k*-mers frequency tables, (2) OTU tables, (3) amplicon sequence variants (via Dada2) tables [53], and (4) pretrained word2vec model-based data [38]. In Table 2, we compare the training data set size’s effect on the models’ accuracies (all Read2Pheno models are trained for 10 epochs and followed by various sample-level classification strategies for sample-level predictions). For example, for the training set of size of 50 samples, our method was trained for 10 epochs with 50 samples and tested by 755 samples, whereas a Random Forest classifier with 100 estimators was trained by the same 50 training samples and tested by 755 samples using the 9-mer frequency feature table, OTU table and ASV table respectively. We use a 9-mer frequency feature table because the filter window size of our convolutional block is 9. As expected, adding more training data increases performance. While training an Random Forest model on raw 9-mers performs very well for all training sizes, our sample embedding and Pseudo OTU methods outperform the 97% identity OTU tables. Moreover, prediction accuracies obtained using the Pseudo OTU approach are competitive with using raw 9-mer features. Finally, the pretrained word2vec model-based method performs comparably to our proposed methods suggesting that our proposed model can learn meaningful embeddings from the experimental data.

**Table 2:** Comparison of sample-level classification accuracy on AGP data. Unlike sample-level classification methods that use OTU/ASV tables and *k*-mers (e.g. 9-mers) as features, our proposed model is trained on reads. Then, read-level results are fused by the sample-level predictor using three methods as described in this paper. By increasing the number of samples in the training data, we compare the read-level classifier’s ability to learn sample-level predictive taxa/information from limited data sizes. The training set size refers to the number of samples used for training. For each training set size, we train 5 different models on 5 sets of randomly selected training samples and the accuracies are averaged and standard deviation is measured over 5 different experiments. We show sample-level prediction for the proposed methods are competitive with prediction from OTU tables and will allow interpretable representations shown in subsequent sections.

		Training Set Size (samples)		
Category	Method	50	400	750
Traditional	9-mer table	0.808 (± 0.039)	0.887 (± 0.009)	0.903 (± 0.024)
	OTU table	0.731 (± 0.021)	0.816 (± 0.010)	0.831 (± 0.030)
	ASV table	0.769 (± 0.034)	0.840 (± 0.014)	0.866 (± 0.024)
Pretrained Embedding [38]	Sample embedding	0.661 (± 0.028)	0.751 (± 0.017)	0.760 (± 0.020)
	Pseudo OTU	0.800 (± 0.018)	0.872 (± 0.012)	0.892 (± 0.019)
Proposed	Majority vote	0.730 (± 0.040)	0.794 (± 0.012)	0.795 (± 0.030)
	Sample embedding	0.751 (± 0.012)	0.816 (± 0.010)	0.821 (± 0.025)
	Pseudo OTU	0.784 (± 0.039)	0.858 (± 0.013)	0.881 (± 0.026)

We also explore a potential way to qualitatively explore how the Pseudo OTU behaves compared with a sequence similarity based clustering method, CD-HIT. We use CD-HIT [59] with 97% similarity threshold to cluster all the training reads of American Gut data set (50 samples). We then compare Pseudo OTU cluster assignment with the CD-HIT cluster assignment results using clustering metrics, homogeneity and completeness [60]. In our experimental setup, a Pseudo OTU clustering result is determined to satisfy homogeneity if all of its clusters contain only data points which are members of a single CD-HIT cluster. A Pseudo OTU clustering result satisfies completeness if all the data points that are members of a given CD-HIT cluster are elements of the same Pseudo OTU cluster. We found that Pseudo OTU achieves 0.787 and 0.794 homogeneity and completeness when compared with CD-HIT’s clustering results. The adjusted Rand index (ARI) [61] for the comparison was found to be 0.519 (values may range from -1 to 1, with 0 being chance). Given that the aforementioned are strict metrics for similarity, this result shows that the embedding vectors do encode read similarity information and can help k-means to create meaningful clusters. In other words, we observe that each read’s embedded vector will embed with similar sequences from the same OTU. As a result, our procedure to validate Pseudo OTU clusters may be adapted to identify the optimal number of clusters required to capture the underlying biological signal.

#### Read embedding visualization

**Fig 2:**
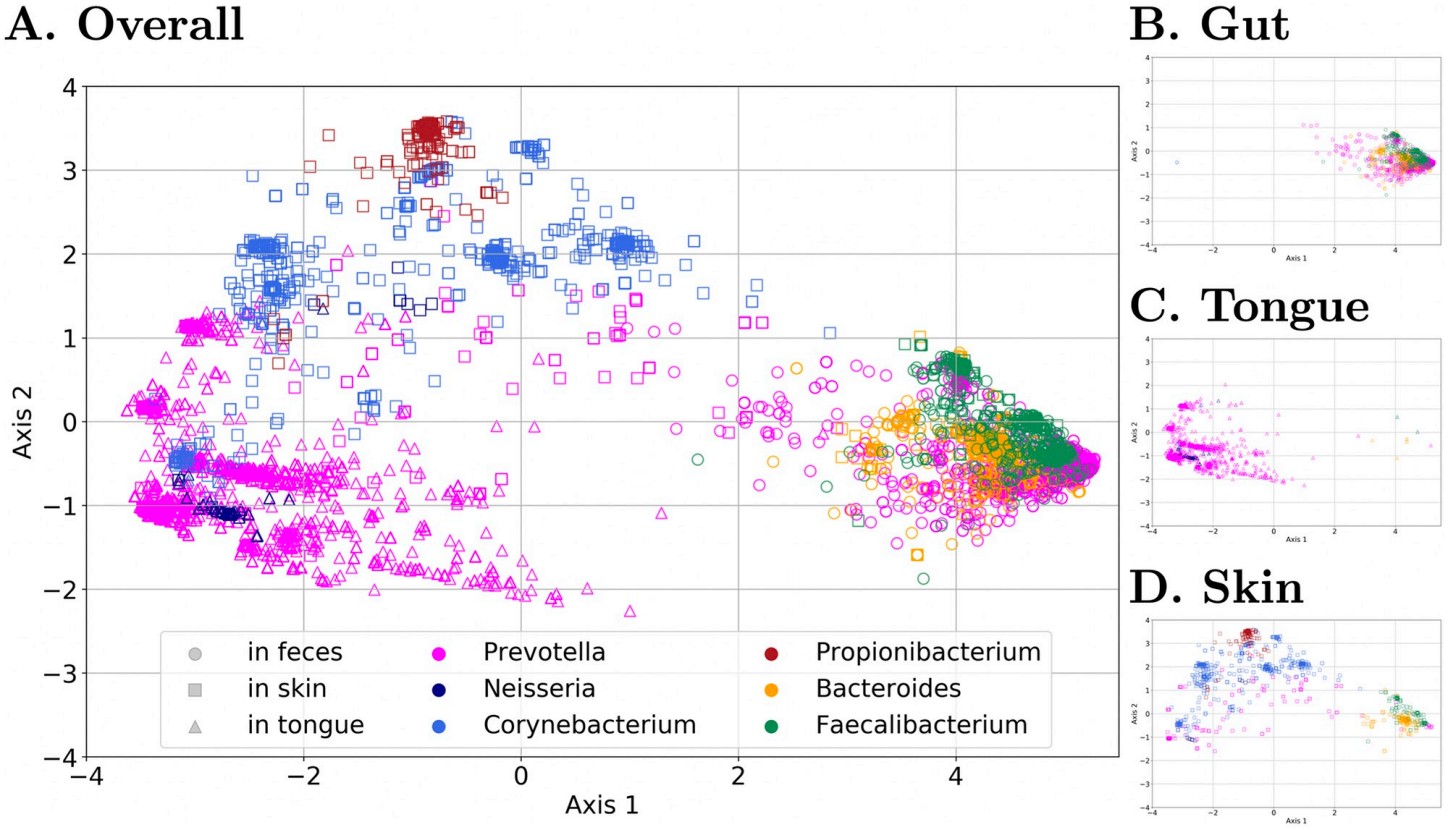
2-D PCA projection of embedded read vectors from all body sites (A), gut (B), tongue (C) and skin (D). ‘▫’s are reads from the skin, ‘△’s are reads from the tongue and ‘°’s are reads from the gut/stool (only a selected set of genera are plotted in the figure). The percentage of the variance explained by the first 2 axes is 58.9%. The different body sites generally cluster closer together and genera/subgenera are more likely to cluster. However, there is a *Prevotella* found in all three body sites, indicating *Prevotella* is found in these three areas (while attention analysis indicates different subgenera are found in each body site). The neural network is learning the 16S rRNA gene association to taxonomy and body site without the access to taxonomy label of the reads.

To illustrate how the Read2Pheno model can learn taxonomic classes despite only having phenotype labels, we visualize the embedded vectors for reads from six selected genera. Fig 2 shows the 2-D principal component analysis (PCA) projection of embedded vectors of 16S rRNA reads (the intermediate output vectors of the Multiplication Layer in Fig 1) of 6 genera from the American Gut data. Each point represents a read, in which the color represents the genus label (determined by RDP [55]) and marker shape represents the original body site (as determined from the body site label). As mentioned in Section Experimental setup for American Gut Project data set and Gevers data set, to produce a clear visualization, we merge skin of hand, skin of head and nostril to one single skin class.

In Fig 2, reads from one body site are clustered closer together than to other body sites in the embedding space. For example, as shown in Fig 2B, reads from the gut are embedded together. In addition, most of the reads from particular genera are closely embedded together. This illustrates that, even though the model is not optimized for taxonomic classification, the neural network is still learning the 16S rRNA variable V4 region—which contains mutations that indicate different taxa—of the input reads in the embedding space. Notably, within the genus *Prevotella*, most fecal-associated reads separate from the oral-associated ones, demonstrating that the model can discern sub-genera which likely prefer different body sites. This kind of intra-genus separation does not appear for genera in which some species/strains occur in multiple body sites. In such cases, the same 16S rRNA read will be found in samples from different body sites, and the model, which predicts a single body site, will fail to predict the read correctly. However, for example in the case of *Corynebacterium*, some skin-associated strains do separate out. Other *Corynebacterium* variants do not separate out, which may indicate that they are associated with multiple body sites.

#### *Prevotella* case study

To understand which features facilitate class separation, we again inspected the read embedded vectors for genera which separated well for the body site isolation source. In Fig 2, reads from *Prevotella* formed two major clusters corresponding to two body sites, namely, tongue and feces. (A 2-D PCA projection showing only *Prevotella* is shown in S12 Appendix). Overall, *Prevotella* test reads were classified to the correct body site source with 91.31% accuracy (balanced accuracy for three body sites is 75.74%). Therefore, we analyze *Prevotella* as an exemplary demonstration of the interpretability of the attention learning mechanism.

**Fig 3:**
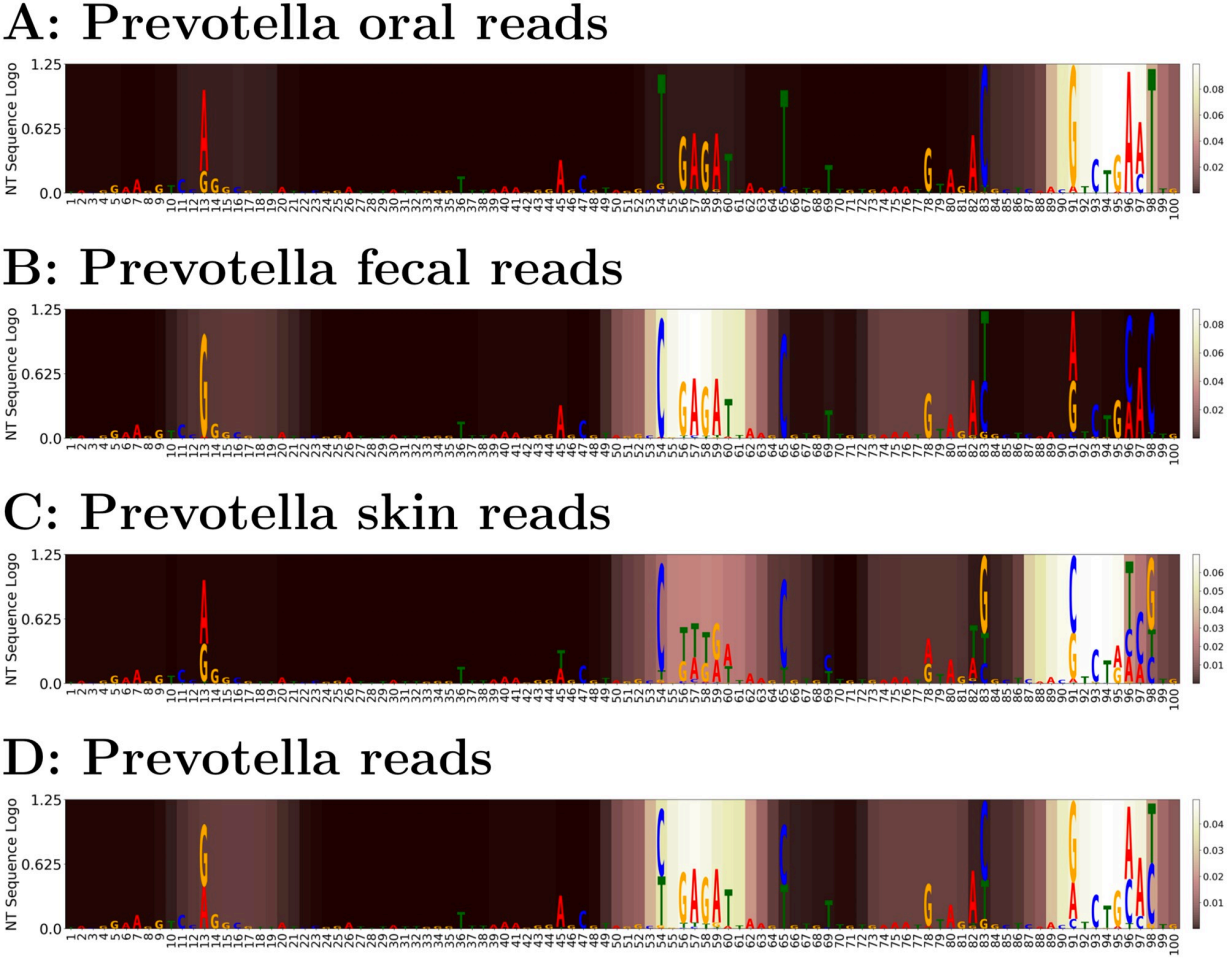
Comparison between average *Prevotella* reads attention and nucleotide frequency entropy in form of nucleotide sequence logo. A: oral reads; B: fecal reads; C: skin reads; D: overall attention. In each body site, nucleotide frequencies are scaled by the overall entropy for all *Prevotella* testing reads and plotted as a sequence logo, with average attention weights represented by a color map where lighter background shading represents larger values for attention weights, in contrast with darker background shading for smaller attention weights. Attention tends to be paid to locations that have distinct sequential information that distinguish body sites. Sequence locations which differ but do not have attention may be important to taxonomic or other information.

To visualize the regions that are most informative to this classification, Fig 3 shows which high entropy positions also have high attention using the method described in sample-level predictors section. Panel D of Fig 3 shows that the middle and end portions of the 100 bp trimmed reads are most important for phenotype classification, with the former playing a more important role in distinguishing fecal reads (panel B) while the latter is more important for oral and skin reads (panels A and C). For visualization, the attention weights are smoothed by a moving average of window size of 9 (i.e., the size used in the convolutional filter of the model). A non-smoothed version of Fig 3 is available in S13 Appendix.

Fig 3 shows the output of the attention layer for *Prevotella* and indicates the areas of the 16S rRNA V4 region to which the network is paying attention for classification (displayed here by the brightness of the highlight). We can see that the end of the 16S V4 region is the most important for identifying oral and skin reads, while the middle region is the most important for gut/fecal reads. However, there are slight differences. For example, an area at the beginning of the V4 region has some importance to also help identify the gut, and to some extent, oral reads, as opposed to finding no attention weighting for skin reads. When comparing oral and skin reads, the middle region is the second most important to identify skin reads. The middle region may help resolve oral/skin body sites that have similar nucleotides at the end of the reads.

For comparison, the entropy of the sequences within the *Prevotella* body site combinations are shown (with the whole general attention shown in Fig 3D). We can see that the attention model generally learns areas of the variable region that have high entropy. However, the model also learns slight differences between the signatures of these regions. For example, both gut/skin reads tend to have C’s located at positions 54 and 65 while gut/oral reads tend to have GAGA at 56 → 59. Thus, the particular combination of C-GAGA—–C is unique to gut reads and, in turn, a high attention weight is placed on this region to distinguish gut reads from other body sites.

#### Comparison of attention-based modeling and *Oligotyping* analysis for *Prevotella* data

To further show how the attention weights based model interpretation compares to other related methods, we run the Oligotyping [41] software package on our *Prevotella* visualization set used in Section *Prevotella* case study. *Oligotyping* uses Shannon entropy to analyze closely related 16S rRNA sequences and find mutations that best explain sample variables (e.g. phenotypes). S14 Appendix shows the oligotypes (highlighted in black) found by their software for the *Prevotella* reads we derived from the AGP data set. As shown in S14 Appendix, in general, the highlighted attention regions are slightly offset or in between high-entropy positions. Regions at or near high-density high-entropy positions (i.e., regions that have many high-entropy positions) are weighted with higher attention. Thus, we infer that there is an apparent relationship between the attention that the neural network learns and the highest entropy positions that are identified. For each body site, there are multiple oligotypes per body site, making it is hard to discern which high entropy position is most important to identifying which body site.

Several factors contribute to the discrepancy between our model’s high attention nucleotide positions and the highest entropy positions used for oligotyping. First, we smooth the attention weights with a 9-mer moving average (because the convolutional filters are are in the window size of 9). As a result, the attention weights are a regional approximation, i.e. not at precise positions, and the weights taper off at the ends of the read, due to edge convolutional effects. Second, *Oligotyping* only calculates the entropy of each position in *Prevotella* reads, while the attention weights learn the weighting of attention of positions that are *specifically* important to the classification task—in this case, body site prediction.

One contrast between *Oligotyping* and Read2Pheno in practice is that, when using *Oligotyping*, a user must plot the distribution of different oligotypes for each phenotype to see if there is a common oligotype for that phenotype, e.g., as in S15 Appendix. From S15 Appendix, the gut (fecal) oligotypes are evidently distinct from the other body sites, while the oral and skin oligotype distributions are relatively similar. Therefore, gut sites could be distinguished from other sites with oligotypes, but more nucleotides would be needed to discern oral/skin. By contrast, using Read2Phone, we found that highlighted attention maps to 4 sequence regions: nucleotide position ranges 12–18, 53–62, 74–81, and 90–98. Whereas region 4 (nt 90–98) helps identify oral reads and skin reads as opposed to gut, regions 1, 2, and 3 are helpful in distinguishing gut samples from other samples. Each body site may be identified by a combination of regions. For example, regions 2, 3, and 4 are useful to identify that a sample was taken from skin, while regions 1 and 4 are most useful for identifying samples from the oral cavity, and 1 and 3 are most useful for identifying gut samples.

### Host phenotype (clinical diagnosis) prediction based on Gevers inflammatory bowel disease data

We further evaluate Read2Pheno performance on a distinct set of sequence data, the Gevers *et al.* data set, which is a subset of data from an inflammatory bowel disease (IBD) study in which samples were identified as being from patients who were diagnosed with inflammatory bowel disease (IBD) and not [5] as described in Section Data preparation for model evaluation. The Gevers *et al.* data set has shown to be consistently challenging to classify. For example, a meta-analysis by Duvallet *et al.* showed that it was one of two IBD studies which fell under 70% AUC [62]. While the accuracy we obtain can range from the high 60%’s by training on a fraction of the data, accuracy is not the ultimate objective of these phenotype prediction experiments.

#### Sample-level attribute prediction

Sample-level attribute, in this case, host phenotype (disease status), prediction is accomplished by 1) the sample-level predictors discussed in S2 Appendix with Read Caller threshold of 0.5 ($\frac{1}{N}$, where *N* = 2 for two classes); 2) sample-level embedding based Random Forest; 3) a Pseudo OTU table based on a Random Forest trained on 1000 Pseudo OTUs. Table 3 compares the accuracy of our model against three baseline methods, a Random Forest classifier trained on 1) *k*-mers, 2) OTU tables, 3) amplicon sequence variants (via Dada2), and (4) pretrained word2vec model-based data. We show the testing accuracy for different training data sizes in Table 3. For example, for the training set size of 40 samples, we trained our method for 10 epochs, and compared that to training a Random Forest classifier with 100 estimators using the various baseline methods. We compare against a Random Forest classifier trained on the 9-mer frequency feature table. *K* = 9 seems like a reasonable choice because the filter window size of our convolutional block is 9. The target classes in this comparison are 2 states: IBD and Non-IBD. From the table, we can see the performance of our Pseudo OTU based method is the comparable to competing methods. When trained with 40 samples and tested on the rest, 9-mer table based model works the best and our Pseudo OTU model is comparable to the OTU table based method. As more samples are used for training, the performances of all models are improved. In general, among our proposed models, the Pseudo OTU model consistently works the best. The Pseudo OTU model is often comparable to OTU and ASV based model but slightly underperforms 9-mer table based model. Similar to the results in Table 2, our embeddings can produce performance on the sample level comparable to the pretrained word2vec model-based method.

**Table 3:** Comparing sample-level prediction accuracy on Gevers et al. Data given increasing training sizes. Unlike OTU/*k*-mer based classifiers, which are trained at sample-level, our proposed model is trained at the read level before read-level results are then fused by sample-level predictor. This comparison, over 40, 160, and 400 samples in the training data shows that the read-level classifier learns predictive taxa/information and the sample-level prediction for the proposed methods are competitive with prediction from OTU tables and will allow interpretable representations shown in the subsequent sections. The obtained accuracy values are averaged, and the standard deviation is computed, over 5 experiments in which we randomly selected training-testing data splits with replacement.

		Training Set Size (samples)		
Category	Method	40	160	400
Traditional	9-mer table	0.715 (± 0.029)	0.787 (± 0.030)	0.848 (± 0.055)
	OTU table	0.684 (± 0.016)	0.768 (± 0.018)	0.843 (± 0.055)
	ASV table	0.669 (± 0.019)	0.765 (± 0.022)	0.819 (± 0.078)
Pretrained Embedding [38]	Sample embedding	0.648 (± 0.036)	0.685 (± 0.020)	0.733 (± 0.057)
	Pseudo OTU	0.684 (± 0.019)	0.762 (± 0.017)	0.843 (± 0.080)
Proposed	Majority vote	0.653 (± 0.043)	0.690 (± 0.023)	0.729 (± 0.062)
	Sample embedding	0.650 (± 0.016)	0.726 (± 0.029)	0.762 (± 0.069)
	Pseudo OTU	0.689 (± 0.031)	0.779 (± 0.014)	0.833 (± 0.058)

#### Read embedding visualization

**Fig 4:**
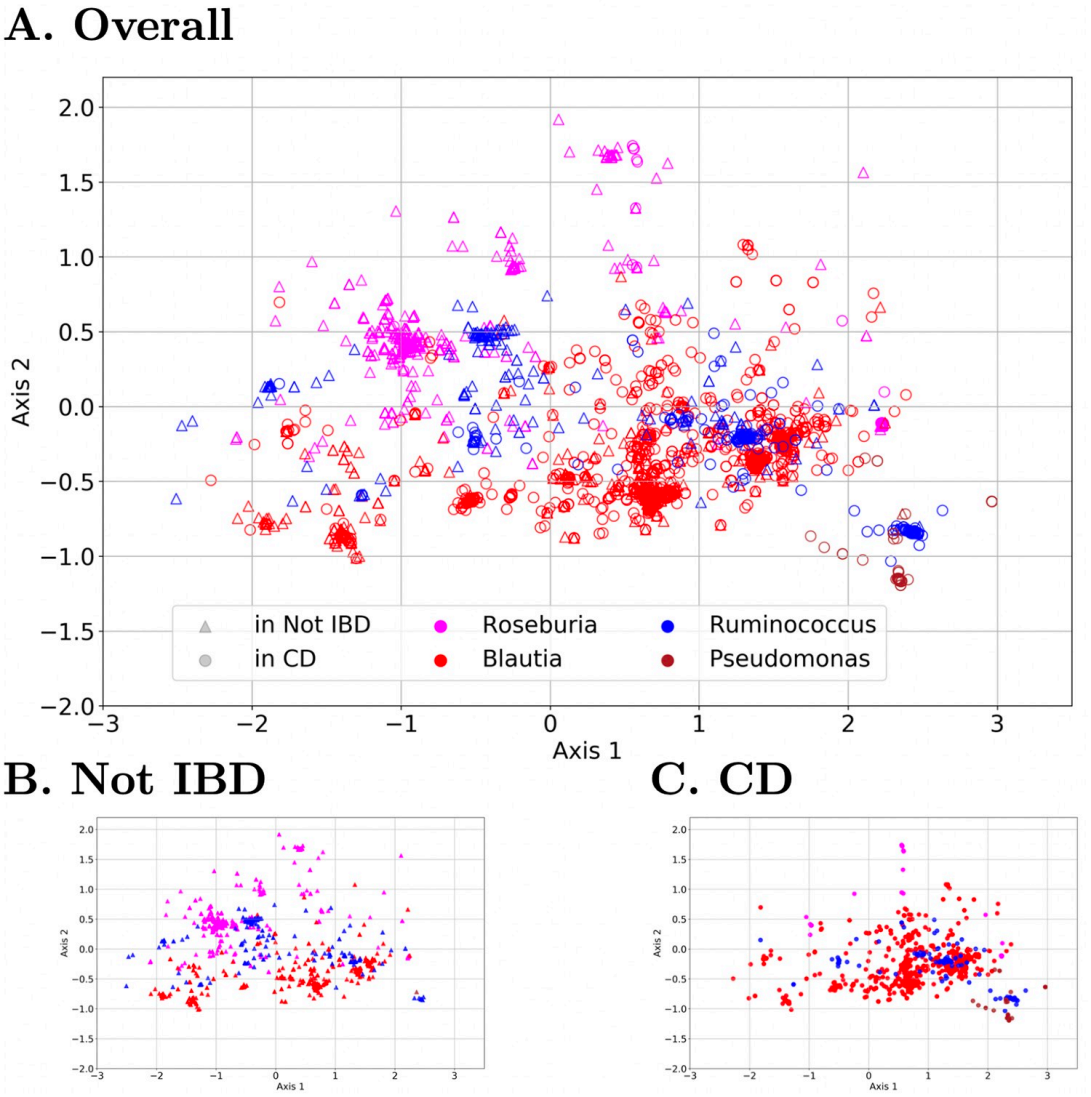
2-D PCA projection of embedded read vectors from all visualization samples (A), disease-positive samples (B), and disease-negative samples (only a selected set of genera are plotted in the figure). ‘△’s are reads from the control (health) samples and ‘°’s are reads from the Crohn’s disease samples. The percentage of the variance explained by the first 2 axes is 52.1%. Compared with Fig 2, the health status and taxonomy are not as tightly clustered demonstrating that while the network is learning this information, it is not as clear (on the first two principal axes).

To inspect how the read embeddings identified by Read2Pheno perform, here we visualize the embedded vectors for reads from 4 selected genera (*Blautia*, *Roseburia*, *Ruminococcus*, and *Pseudomonas*). Fig 4 shows the 2-D PCA projection of embedded vectors (the intermediate output vectors of the multiplication layer in Fig 1) of a selection of 4 genera from 16S rRNA reads from the Gevers Crohn’s disease data set [5]. Each point represents a read, in which the color represents the genus label (determined by RDP [55]) and marker shape represents the disease state (CD: Crohn’s disease; Not IBD: No inflammatory bowel disease diagnosis).

In Fig 4, reads from one genus are closely embedded together most of the time. However, the disease-negative (“Not IBD”) samples for the *Roseburia* and *Ruminococcus* genera have the widest spread in PCA. In fact, we can see multiple clusters in most of the genera, suggesting that different sub-genera cluster together and can be associated with different phenotypes. In addition, reads identified as disease-positive (“CD”) are generally clustered at the lower right of the figure, while reads labeled as disease-negative are clustered at the upper left. Even though the model has not been trained to do taxonomic classification—only disease phenotype—it can still reflect what it has learned from the sequence structure of the input reads in the embedding space to reveal taxonomic structure.

Indeed, within *Blautia* and *Ruminococcus* genera, there are at least one cluster corresponding to disease-positive and another cluster corresponding to disease-negative (see S17 Appendix). Finally, most *Pseudomonas* reads are disease-positive, and they are also embedded in lower right corner in the figure, which shows that the Read2Pheno model has predicted that those reads are disease-positive. This is consistent with the association between *Pseudomonas* species and IBD, which has been described extensively in the literature [63–65].

#### *Blautia* case study

**Fig 5:**
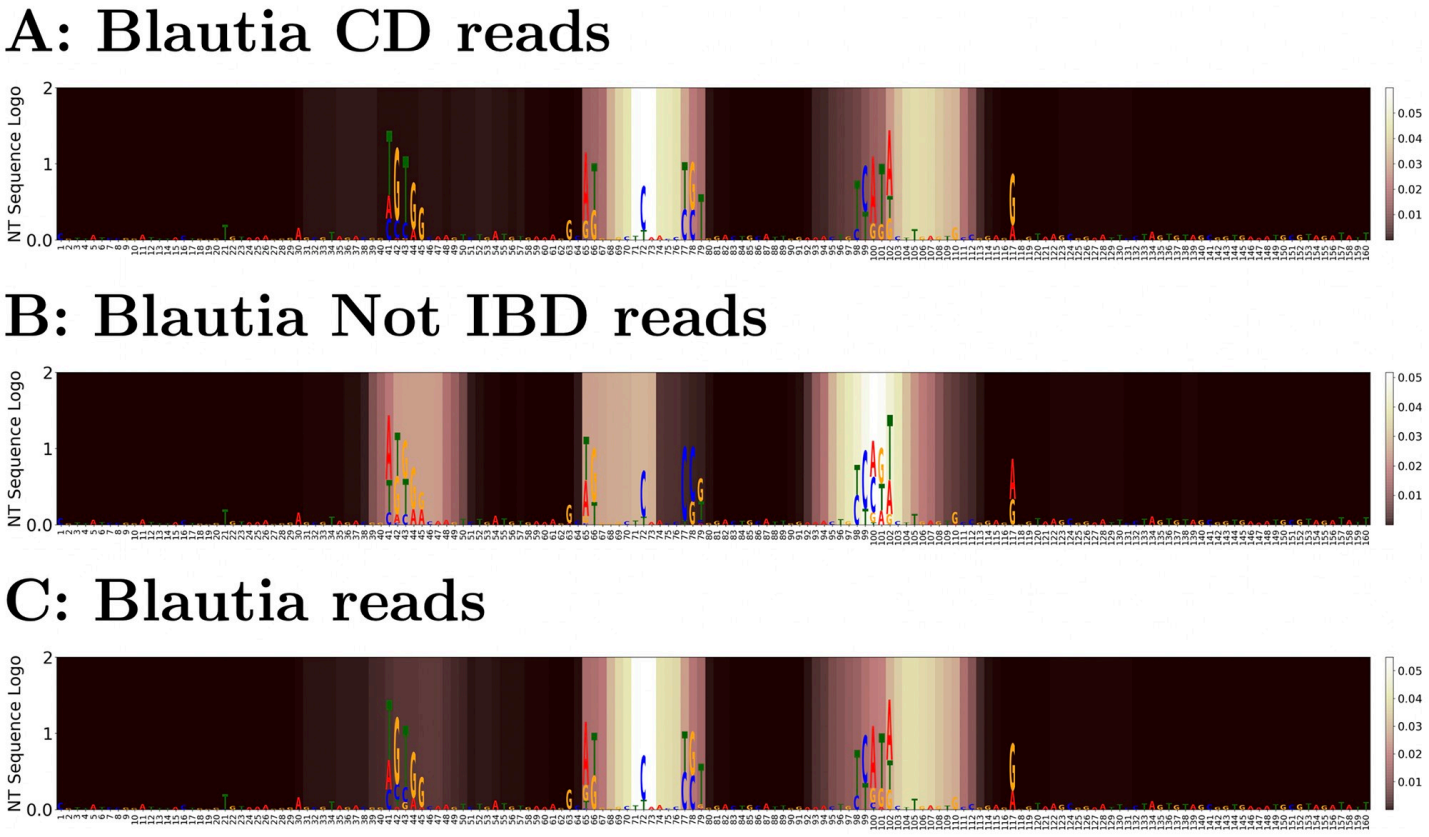
Comparison between average *Blautia* reads attention and nucleotide frequency entropy in form of nucleotide sequence logo. A: “CD” reads; B: “Not IBD” reads; C: overall attention. In each phenotype, nucleotide frequencies are scaled by the overall entropy for all *Blautia* testing reads and plotted as sequence logo and averaged attention weights are represented by the color map (brighter colors represent larger attention weights). Two areas have attention that differentiate *Blautia* in Crohn’s Disease from *Blautia* in Non-IBD, while a third site is important for identification of *Blautia* subgenera associated with non-IBD.

We further analyze the interpretability of our model by inspecting the attention weights in Fig 5. The *Blautia* test reads were classified with 73.62% accuracy (with a balanced accuracy of 39.52% because this model has high false positive rate on this genus). As shown in the figure, the high attention weight region and the high nucleotide variance region coincides well with each other. That is to say, the model pays attention to nucleotide variable regions which are informative to distinguish *Blautia* reads founds in disease-positive and -negative samples.

### Taxonomy prediction based on SILVA full length 16S rRNA sequence data

We further evaluate the capability of our proposed Read2Pheno model to analyze and learn which regions of the full-length 16S ribosomal RNA sequence are useful for predicting the genus level taxonomic label. The experimental data set in this section is constructed from the SILVA 16S ribosomal RNA gene database [50] and their manually curated taxonomy [51] (i.e., Release 132 16S sequences with 99% identity criterion to remove highly identical sequences).

#### Full length 16S rRNA taxonomic classification

Unlike the other results presented above, here, we train our proposed Read2Pheno model on *taxonomic* classification specifically. In particular, we train the model on the SILVA training data set for 40 epochs. We adopt the same set of parameters and NN model architecture used in the previous experiments (256 filters in CNN layers, 64 units in LSTM layer, 0 dropout rate and 0.001 learning rate), except for the number of neurons in output node, *N*_*y*_, which must be set to 495 to accommodate all the genera classes. The same training data are used to train the QIIME [54] implementation of RDP [55] taxonomic classifier. Then, both the RDP classifier and our Read2Pheno model are tested by the testing data set. Table 4 shows the results of both models.

**Table 4:** Accuracy comparison on the SILVA data set over 5-fold cross-validation. The proposed model’s performance is slightly below but still competitive to RDP’s accuracy.

Method	Avg. Accuracy (std)
RDP implemented in QIIME	0.976 (± 0.001)
Proposed model	0.959 (± 0.006)

#### Full length 16S rRNA sequence visualization

**Fig 6:**
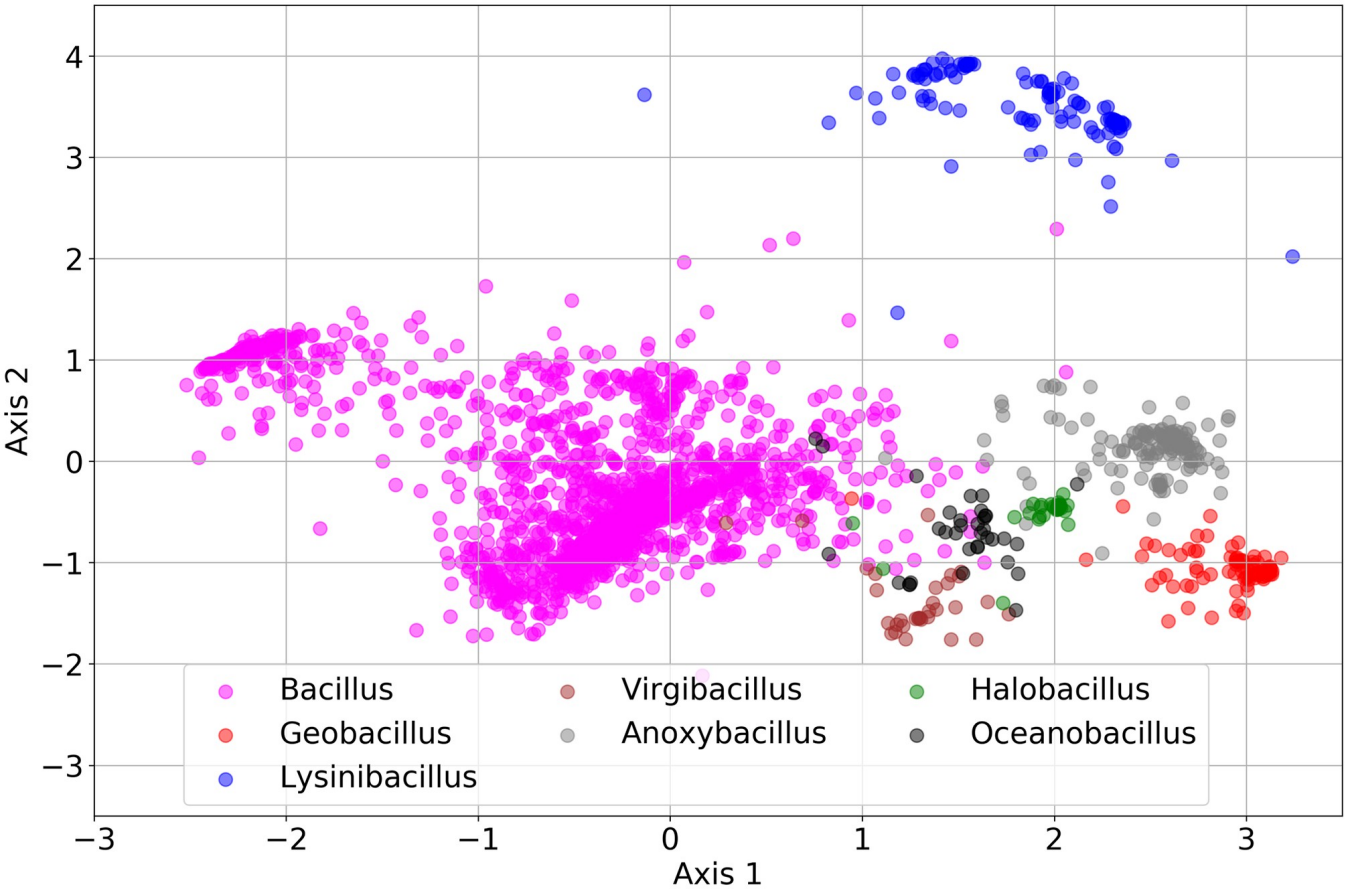
2-D PCA projection of embedded testing sequence vectors from 7 selected genera in the *Bacillaceae* family. The percentage of the variance explained by the first 2 axes is 42.7%. The model can separate sequences with respect to their genus level label based on their genomic content.

We visualize the embedded vectors for full-length 16S rRNA sequences from 7 selected genera in the *Bacillaceae* family in Fig 6. A 2-D principal component analysis (PCA) projects the embedded vectors (the intermediate output vectors of the Multiplication Layer in Fig 1) of the sequences from the selected 7 genera. Each point represents a sequence, in which the color represents the genus label. In Fig 6, most of the sequences from one genus are closely embedded together and sequences from different genera are embedded apart from each other. This illustrates that the model can learn taxonomic information directly from labels. (Above, we showed the model learning taxonomic information indirectly from phenotype labels in the AGP data set.) Although distorted from the 2-D projection, we can also see that some *Bacilli* have 16S rRNA sequences which may be similar to other types of *Bacilli* genera like *Virgi-* and *Oceano-bacillus* but be more distinct from *Geobacillus*. This could indicate misclassifications of these sequences in the standard taxonomy (e.g. as found in Bergey’s Manual of Systematic Bacteriology) or simply evolutionary relatedness between taxa.

#### Discovery and analysis of variable regions by attention weights in *Pseudomonas* and *Enterobacter*

**Fig 7:**
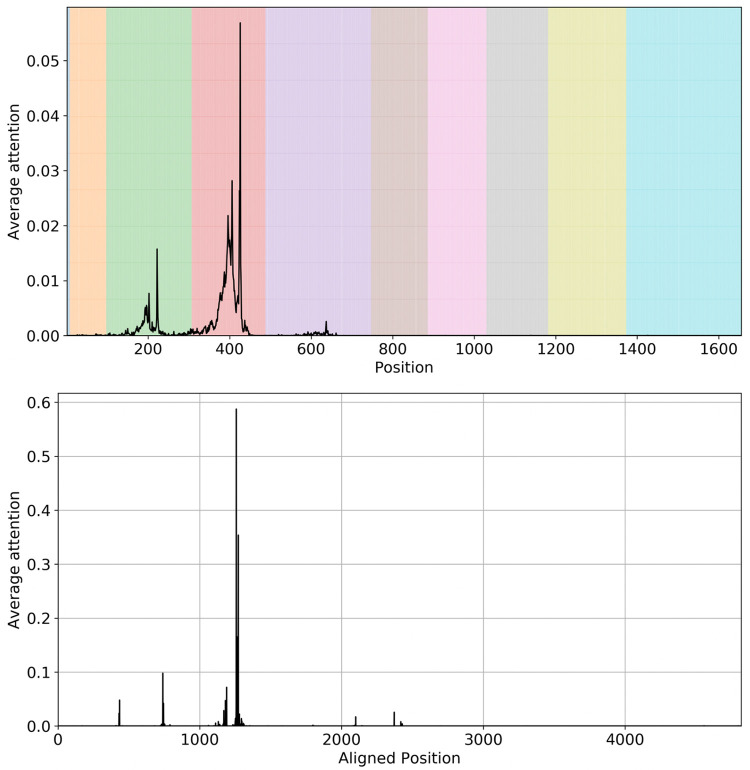
Top: Average attention weights for all strains of *Pseudomonas* without alignment; Bottom: Average of attention weights for all strains with alignment. The color indicates the variable regions break points [66]. This figure shows that our model can implicitly learn the multiple sequence alignment. In particular, the model learned to pay attention to important regions. Before alignment, attention is paid to a wider region of positions. However, after sequence alignment, attention weights are concentrated among a smaller range of positions (i.e., where the maximum averaged attention weight is more than 10 times higher than the weight pre-alignment). This indicates that the model can recognize certain nucleotide configurations and is robust to insertions and deletions that would be accounted for by the sequence alignment.

In Fig 7 and S18 Appendix, we visualize the average attention weights of testing sequences from two selected genera. For *Pseudomonas* in Fig 7, the top figure shows the averaged attention weights per all testing sequences without alignment. The variable regions are labeled according to [66]. Here, different colors correspond to different variable region (from V1 to V9 as shown in the colorbar). As we can see, the attention weights highlight nucleotides concentrated on V2 and V3 regions. There are insertion and deletions in different *Pseudomonas* sequences. As a result, the location of a certain sequence context that gains high attention weights can be shifted in different sequences. We applied multiple sequence alignment to align the testing sequences for *Pseudomonas* using the MAFFT on XSEDE [67]. The attention weights are then aligned by the sequence alignment results. Then, the average attention are computed based on these aligned attention vectors. From the bottom figure in Fig 7, we observe that the attention sites narrow down to a few select nucleotide positions despite insertions and deletions in the 16S rRNA evolution. This is evidence that our model is learning specific 16S rRNA nucleotide contexts that are important to the distinction of taxa and decides where to pay the attention based on the context. We further visualize the attention weights of a real *Pseudomonas aeruginosa* 16s rRNA sequence found in [68] in S19 Appendix. We analyzed the positions that have an attention weight greater than the mean attention weight across the whole sequence. The attention-highlighted regions coincide with sub-regions of V2, V3, and V4 variable regions determined by [68]. Moreover, analysis of the weights shows that the model pays attention to certain junctions in the rRNA secondary structure. We conjecture that those junctions may be related to molecular interactions, and that the nucleotides at those positions may contribute to the angle/structure of the arms associated with the junction.

We also applied the same analysis on *Enterobacter* as a comparison (result shown in S18 Appendix). For *Enterobacter*, the attention weights are concentrated on V2 and V4. After multiple sequence alignment, we observe concentration of attention weights into mainly two sites (similar narrowing trend as in *Pseudomonas*), which implies that the model can learn contextual information and is robust to insertions/deletions/mutations. Full multiple sequence alignment results in FASTA format and the mean attention weights per position after alignment are available in S20 Appendix. To offer insight into what the neural network is learning as the most important variable regions for each genus, we calculated the sum of attention weights of testing sequences per variable region (with variable region locations defined by [66]) for each genus and can be found in S21 Appendix. There has been previous work that aimed to find important/predictive variable regions that can inform taxonomic classification and V2, V3, V4 are considered as “informative” [69–72]. Our model, in most cases, pays most attention to the V3 and V4 regions, which many studies now use as common targets. Genera such as *Buchnera*, *Erwinia*, and *Gemmata* have higher attention weights on the V2 region.

### Computational performance of Read2Pheno training

We used the GPU node on the Extreme Science and Engineering Discovery Environment (XSEDE) computing resource [56] with one P100 GPU to train our model. It took approximately 0.89, 1.58, 9.15 hours to train the models for the American Gut (50 samples), the Gevers *et al.* (40 samples) and the SILVA data sets respectively. The memory consumption is proportional to the number of training samples. For example, 7.1 GB memory was used when training the model on the American Gut (50 samples) data set (Constructing an OTU table on the same data set took 0.49 hours and 11.7 GB memory). When we trained the model on a 750 samples from American Gut data set, it required over 74 GB memory and took 13.83 hours to complete the training process. However, as discussed above, the Read2Pheno model may be effectively trained with smaller data sets. In particular, for the American Gut data set, robust performance was found when trained on 50 samples representing over 1 million reads. We further note that the use of smaller batch size and more efficient data structures than those utilized in our code can address additional memory demands, for example as the read length increases.

## Discussion

We have shown that our proposed attention-based deep neural network model for read-level classification, namely Read2Pheno models, are capable of comparable accuracy prediction performance while offering automated model interpretation on three distinct kinds of tasks: (1) prediction of microbiome phenotype (i.e., the emergent property of a microbial community), (2) prediction of host phenotype (i.e., clinical disease diagnosis), and (3) taxonomic classification of full length 16S rRNA sequences. The implications of our attention-based deep learning methodology, as implemented and evaluated on these tasks as proof-of-concept, are discussed in further detail below.

As proof-of-concept for microbiome and phenotype prediction, we have focused on two large-scale microbiome data sets. *First*, we have analyzed data from the American Gut Project, which provides a comprehensive open-source and open-access set of human microbiome 16S rRNA samples for scientific use [49, 73]. The recent studies of microbiomes inhabiting sites on the human body (particularly the large intestine) have revealed the complex nature of microbial community interactions [73]. 16S ribosomal RNA is not only useful for identifying organisms using the phylogenetic tree of life, but the phylogenetic branch distance shared between samples serves as a comparative distance metric [74]. Utilizing the AGP’s large collection of samples, where we can identify organisms via 16S rRNA, allows us to begin to understand microbial community dynamics in hosts and the environment [75]. The AGP data set thus provides real-world data to develop and validate phenotype prediction algorithms. *Second*, to analyze host phenotype, we have further looked to gut data with clinical significance as well. Crohn’s disease (CD), a chronic relapsing inflammatory bowel disease (IBD), is increasing in prevalence worldwide [76]. Researchers have been exploring different methods to predict Crohn’s disease based using microbiome data, for example to identify the microbial taxa that associated with the disease using 16S rRNA survey data [5, 7, 77]. We have further evaluated Read2Pheno and sample-level classification using a data set based on clinical evaluation provided by Gevers *et al.* Developing a better understanding of *the* IBD microbial signature will present a critical step towards improved clinical diagnosis and discovery of a cure.

Overall, our results show that we can keep both local information (*k*-mers) and contextual information (sequential order of *k*-mers) of 16S rRNA regions without having to preprocess the data to create abundance tables, such as OTU or ASV tables, for phenotype prediction. Doing so, we can still achieve comparable performance to phenotype classification based on OTUs/ASVs. As we have demonstrated, the key advantage of this approach is that we can highlight informative regions (i.e., regions that are material for classification) within sequences. There are potentially other advantages as well of our read-level approach. Using aggregated OTU or ASV tables, i.e., sample-level prediction, constrains the size of a training set for a complex deep neural network to the number of samples–which will almost always be so relatively small as to risk overfitting (especially when the variation in the data set is low) [78, 79]. For example, there are over 1 million parameters in the proposed deep neural network model for AGP experimental data, but there are only 805 samples (when perfectly balanced). By contrast, to avoid overfitting the number of data points, *n*, should be no less than some multiple (say 5 or 10) of the number of adaptive parameters in the model [78, 79]. Indeed, even when we consider all 15,096 samples in the American Gut Project data set as of May 2017, which mostly were collected from feces, the number of training samples is still less than $\frac{1}{100}$ of the total number of trainable parameters, *m*.

Given the limitations on sample collection, a read-level model like Read2Pheno advantageously allows one to leverage up to millions of reads as training examples from only dozens of samples. As shown in Table 2 and 3, the proposed models with different training sizes obtain similar results to standard methods and that overfitting issue is not a concern. While there will be redundant information in reads, and potentially more feature density, it provides a path to avoid overfitting complex deep learning models. A caveat to our read-level prediction approach is that we take a subset of samples in order to generate balanced classes for training. Class imbalance in microbiome data has been recognized as a serious problem for machine learning approaches [39]. In particular, our approach training on imbalanced classes at the read-level can give rise to bias due to potential taxon overrepresentation in the majority class. Notably, we have more than 1 million reads even by only considering a few dozen of samples, which is very different from the scenario for sample-level classification in which researchers look to data augmentation, for example, to create adequately large training sets for training with class imbalances. Indeed, we found that we have ample training data to adequately train the model with those reads as a proof-of-concept in our study, as shown, for example, by our sample-level validation results for the Gevers data set (see, e.g., 3). Although it is outside the scope of this paper, we are also considering experimenting with assigning a class weight to reads in the optimization process.

Sample-level prediction has been used primarily here to validate read-level models. As a result, there is substantial room to improve methods to predict sample-level attributes from read-level models. In this paper, we have considered majority voting, weighted schemes, and averaging embedding weights; there may be other approaches as well. However, as our results for Pseudo OTU classification using random forests demonstrate, conducting sample-level learning based on read-level embeddings may prove to be promising path to sample/community-level prediction. Our experiments showed competitive or superior performance as compared to OTU/ASV tables, which require an initial step of identifying taxonomic composition. The Pseudo OTU approach did not perform significantly worse than *k*-mer table-based classification, which suffers from the difficulty of tying *k*-mer information back to taxonomy or the larger sequence context. Additionally, using clustering based on sequence similarity with CD-HIT for validation, we can show that *k*-means clustering based on the Pseudo OTU creates meaningful and interpretable clusters.

Attention-based deep learning, such as Read2Pheno, for microbiome sample analysis advantageously avoids preprocessing steps needed for conventional methods. For example, unlike conventional OTU-based methods, sample prediction using Read2Pheno does not require an alignment/OTU grouping step. Therefore, our proposed method lowers the risk of introducing sources of error from preprocessing (e.g., due to lack of domain knowledge) and user-dependent choices. Although hyperparameter tuning can play a more important role in deep learning based methods, it can be done programmatically and human intervention can be minimized. Furthermore, the intermediate layer outputs of our Read2Pheno method can be used for ordination and highlight informative regions in the sequences. Visualizing intermediate layer outputs thus supplants post-processing required to analyze the results of OTU-based methods. Therefore, our model eliminates the need for pre- and post-processing associated with conventional OTU-based methods. Instead, our end-to-end model takes in raw 16S rRNA reads as input, learns informative genetic content and label association, and outputs classification results. And, the learned knowledge, i.e. explanation for the model, can be automatically extracted by intermediate layer outputs, in particular the embedding vectors and attention weights. Our model has also proven to be robust to deletions and insertions: as shown in Fig 7, it can implicitly identify relevant genetic content across different unaligned sequences from the same genus).

Notably, the attention-based model highlights regions that greatly influence the model’s decision. Therefore, the interpretation depends on a specific model—and can vary from model to model, even when they are trained on the same task. For example, we show the attention weights plots for body site prediction models (the counterpart of Fig 3) trained by AGP datasets with 400 samples in S22 Appendix and 750 samples in S23 Appendix, and the attention weights plots for Crohn’s disease prediction models (the counterpart of Fig 5) trained by Gevers dataset with 160 samples in S24 Appendix and 400 samples in S25 Appendix. The attention weights depends on both the performance of the trained model and the reads chosen for visualization. As shown in S25 Appendix, the Crohn’s disease prediction model trained on 400 samples achieves the highest classification accuracy on *Blautia* visualization reads. By contrast, the model trained on 160 samples produces the worst Crohn’s disease prediction accuracy on the corresponding *Blautia* visualization reads. When inspecting the attention weights of these models, we can see that the worst-accuracy model pays less attention to the middle variable region (around position 60 to 80) in S24 Appendix, whereas the other better-accuracy models pay more attention to the middle variable region, as shown in both Fig 5 and S25 Appendix. From AGP experimental results in S22 and S23 Appendices, on the other hand, we can see that the attention weights of different models highlight similar regions despite of some variations. Accordingly, the classification accuracy of our model can provide confidence in the importance of the high attention regions.

Because our experimental datasets are 16S rRNA reads/sequences, which are easily aligned, we can also further link the attention weights with entropy to better aid human interpretation of high attention regions. We offer this framework for researchers to gain insight from training and interpreting models. One major contribution of this framework is that, instead of selecting Oligotypes based on a trial-and-error approach, our model can find the most informative sites that discriminate classes. Further, any required hyperparameter searches can be performed systematically since our method employs supervised learning, i.e., the model is being optimized to a defined objective. Oligotyping, on the other hand, is unsupervised, and tuning it requires more human intervention.

The efficiency of our proposed model’s interpretability also contrasts favorably with *Oligotyping*. Again, an attention-based model like Read2Pheno avoids the need for user intervention through pre-processing decisions. As noted above, with *Oligotyping*, one must choose the number of nucleotides to examine—and this choice affects the outcome. To obtain the results reported in this paper, for example, we chose 7 nucleotides, since those represented nucleotide positions from all 4 regions in which we found attention. Moreover, there is no guarantee that *Oligotyping* will be able to succeed at all desired classification/discrimination tasks. For example, as S15 Appendix shows, while the gut has a more distinct pattern of oligotypes as opposed to oral and skin samples, oral and skin samples show very little difference in oligotype patterns. As such, it is practically difficult to perform this common classification task using only learned oligotypes. By contrast, the attention-based Read2Pheno model in this paper are demonstrated to be able to deliver accurate classification results, in addition to the ability to discover sequence subregions which are significant in the determining the classification.

Ideally, we would like to go beyond the qualitative comparisons that we have made here between the entropy logos generated by our attention-based model and Oligotyping. A more rigorous comparison will require the generation of a “ground truth”, where particular strains with certain mutations and their relation to host/environmental phenotype can be experimentally verified. Such a data set would be generally useful for the development and benchmarking of computational approaches [80, 81].

In addition to training and evaluating attention-based models on phenotype data, we have further considered a comprehensive data set for taxonomic classification, the SILVA rRNA database. Applying Read2Pheno to SILVA data demonstrates that it can be used to explore the structure of full length 16S sequences by using such full length sequences to train the model for a taxonomic classification task. (In a sense, this can be thought of as a read2taxa task, but it employs the same Read2Pheno model as used in phenotypic studies). Our model achieves comparable performance to the superior *k*-mer based method and pays attention to different regions for genus classification. This indicates that parts of the 16S rRNA gene, for a given 16S rRNA sequence from a genus, are important for distinguishing one genus from other genera. Notably, *Salmonella* has higher weights on the V2 and V4 regions of the sequence. Identifying high-attention regions can inform future 16S rRNA study designs. For example, because the V2 region is informative (i.e., has higher attention weights), investigators could use that as a basis on focusing on the design and use of primers to target the V2 region to augment more commonly used V3/V4 primers. Some genera, such as *Pirellula*, have attention within some regions (V2 and V4) but also have higher than normal attentions at other sites, which demonstrates that more regions can be used to discern such a genus. The predictions which Read2Pheno was used to make in this paper are consistent with evidence in the experimental literature. In particular, previous studies have shown that the V1-V3 regions are better at distinguishing *Escherichia*/*Shigella* [82], and we show high attention within the V2 and V3 regions for this group. The model also predicts that *Methanobacterium*, *Thermococcus*, and other Archaea have attention weighting at the V4-V5 regions. And, indeed, the V4-V5 regions have already been shown to have superior recognition of *Archaea* [83]. Accordingly, the Read2Pheno model’s predictions can serve as a starting point for identifying which primers may be optimally used to target various genera.

## Conclusion

In this paper we have integrated CNNs, RNNs, and attention mechanisms, to develop a deep learning model, Read2Pheno. The Read2Pheno model can perform alignment-free (and robust to insertion and deletion) microbial DNA sequence analysis to achieve read- and sample-level environmental prediction ***and*** extract interesting sequence features. In particular, we have shown in computational experiments that the Read2Pheno model can accomplish diverse tasks, learning informative nucleotides to (i) predict the body sites from which human microbiome samples are extracted, (ii) discriminate between samples from individuals with positive and negative diagnoses for IBD, and (iii) identify the taxonomic labels of whole sequence 16S rRNA data. Moreover, we have shown that because it exploits attention mechanisms, the Read2Pheno model can not only predict, but can also be readily interpreted to obtain further insight. We have also shown that we can interpret the intermediate outputs in the neural network model and generate visualization of the read embedding vectors. We can thus discover important taxa and regions of sequences that are highly associated with the sample phenotype/taxonomic label. We show that our deep learning model can be used to explore the 16S rRNA nucleotide structure and its association with phenotype and taxonomy by learning the high-level features from *k*-mers and their sequential order.

Our paper further sets forth an alternative way to train deep neural networks for predicting phenotype from microbiome samples when the number of samples are relatively small: first training a read-level classifier, i.e., Read2Pheno, instead of a sample-level classifier—and still producing comparable classification results. Moreover, even in cases where our proposed attention-based neural network modeling framework fails to provide clearly superior classification performance at the sample-level, we demonstrate that the results are nonetheless easy to interpret, particularly when compared to conventional methods for predicting phenotype based on microbiome sequence samples. The Read2Pheno model thus provides an exploratory tool for future microbiome studies, as it can be used to extract important biologically and clinically relevant information from complex sequence data from limited numbers of patient or environmental samples.

## Supporting information

S1 Appendix One-hot coding map. The map is adapted from Nomenclature for Incompletely Specified Bases in Nucleic Acid Sequences (http://www.sbcs.qmul.ac.uk/iubmb/misc/naseq.html).
(XLSX)

S2 AppendixSample-level predictor: Majority vote method.The read caller threshold is $\frac{1}{N}$ where N is the number of classes.(TIF)

S3 Appendix Sample-level predictor: Sample-level embedding. Sample-level embedding is calculated by averaging all the read-level embedding vectors per sample. Then, a random forest classifier is trained based on the sample embedding matrix (an *N* by *N*_*h*_ matrix where *N* is the total number of samples in training set and *N*_*h*_ is the number of hidden nodes in Bi-LSTM layer). Once the sample-level random forest classifier is trained, this model can be used to perform sample-level classification by taking the sample embedding vectors as input. The training and testing process are labeled by red and blue arrows correspondingly.
(TIF)

S4 Appendix Sample-level predictor: Pseudo OTU. Training reads are clustered into *N*_*clusters*_ = 1000 clusters (Pseudo OTUs) by the read-level embedding clustering module using a *k* − *means* algorithm. Then, all training reads per sample are mapped to the closest Pseudo OTUs to form Pseudo OTUs abundance table. Similar to sample-level embedding method, a random forest classifier can be trained to perform sample-level prediction using such Pseudo OTUs table (The training and testing process are labeled by red and blue arrows correspondingly).
(TIF)

S5 Appendix American Gut Project (AGP) metadata. The 805 sample subset of AGP data used for the experiments in this paper were obtained from 421 individuals. We have prepared a supplementary file with the metadata of the AGP experimental data set used in the paper, which provides the age, sex, race, BMI and health status, as well information about the split between training and test data sets.
(CSV)

S6 Appendix Overall training and testing experiment. Samples are split into train and test set. Training set is used to train a Read2Pheno classifier and a sample-level predictor. The testing set is used to evaluate the performance.
(TIF)

S7 Appendix Read2Pheno training process. All reads in the training set are labeled by the sample-level label (the body site the original sample was collected from). Then the reads are grouped together and shuffled for training the Read2Pheno classifier.
(TIF)

S8 Appendix Hyperparameter search space table. The best set of parameters is {number of conv filters, *N*_*c*_: 256, number of units in LSTM *N*_*h*_: 64, dropout probability for Dropout Layer: 0, learning rate: 0.001} for read-level prediction on 5-fold cross validation of training data. The window size of convolutional layers, *W*, is set to 9 and the number of hidden nodes in attention layer, *N*_*a*_, is set to 16 in the default setting. Cole *et al.* found that the Naïve Bayes classifier performs similarly when using 8-mer and 9-mer as features but slightly worse using smaller *k*-mers [84] for the taxonomic classification of 16S rRNA reads. Therefore, we similarly use a window size of 9 for convolutional filters. In S11 Appendix, we further show that sample-level classification performance of the model is generally insensitive to the window size. In future work, we will consider other consequences of altering window size, such as computational performance and interpretability.
(XLSX)

S9 Appendix Training and validation loss as a function of epochs for the Read2Pheno model on American Gut Project data. This figure shows the cross-entropy loss of our model in training and validation as a function of epochs. From this figure, we can see that the validation loss stabilized after epoch number 7.
(TIF)

S10 Appendix Training data size effect of Read2Pheno classifier. To evaluate the influence of the training size for Read2Pheno classifier training, we designed an independent experiment: we first hold out 55 samples for testing, then we train the Read2Pheno model with reads from 5, 25, 50, 100, 500 and 750 samples from the rest of samples and evaluate the sample-level performance of those models by the 55 samples in the held-out testing set. (Here, we use the sample-level embedding method for sample prediction). To determine how much sample-level prediction quality depends on the size of the Read2Pheno training set as compared to the size of the downstream sample-level training (i.e. Random Forest) set, we measure test set prediction accuracy where sample-level Random Forest is trained 1) on the same training set as the Read2Pheno classifier, and 2) on all 750 samples irrespective of the number of samples used to train Read2Pheno. The blue curve shows the training type 1 and the orange line shows the training type 2. The blue curve shows that as more samples used for training, the sample-level accuracy increases. The orange curve shows that although Read2Pheno classifiers are trained with different samples, as long as the sample-level prediction model is trained with more samples, the performance is generally stable.
(TIF)

S11 Appendix Evaluation of sensitivity of Read2Pheno modeling to different window sizes of convolutional layers. In this table, we compare the performance of sample phenotype prediction on American Gut Project data as the window size of convolutional layers is varied (i.e., varying the hyperparameter, *W*, in Fig 1). In this experiment, we evaluated the model with 4 other window sizes, namely, 3, 6, 12 and 15, in addition to our default windows size, 9. We train and evaluate all different models with the 50-sample experimental AGP data set used in Table 2. Similarly, prediction accuracies are averaged and standard deviation is measured over 5 randomly selected data with replacement experiments. The table shows that the sample-level prediction accuracy is generally not sensitive to the window size parameter of the Read2Pheno model.
(PDF)

S12 Appendix 2-D projection of embedded *Prevotella* read vectors. Red markers represent fecal reads, green markers represent oral reads and black markers represent skin reads. The color of a ‘×’ represents the predicted body site. If the predicted body site is the same as the true body site, then ‘×’s are not visible. The body site prediction accuracy for *Prevotella* visualization reads is 0.9131.
(TIF)

S13 Appendix Visualization of the attention weights without smoothing. Comparison between average *Prevotella* reads attention weights and nucleotide frequency entropy in form of nucleotide sequence logo. A: oral reads; B: fecal reads; C: skin reads; D: overall attention. In each body site, nucleotide frequencies are scaled by the overall entropy for all *Prevotella* testing reads and plotted as a sequence logo, with average attention weights represented by a color map where lighter background shading represents larger values for attention weights, in contrast with darker background shading for smaller attention weights. Attention weights are not smoothed by the moving average of window size of 9 as opposed to Fig 3.
(TIF)

S14 Appendix Oligotypes for *Prevotella* reads. Top 7 Oligotypes found in *Prevotella* reads. The number on the left hand side of the figure shows the number of reads having a certain type of *Oligotyping* patterns (the nucleotide combination in black positions).
(TIF)

S15 Appendix Oligotypes and body site association [41] of *Prevotella* reads. Different 7-oligotypes configurations can be found in different body sites. Fecal samples have a distinctive configuration as compared with oral and skin samples. Of note, *Oligotyping* for 7 positions picks positions 13, 54, 65, 83, 91, 96, and 98. In our model, shown in Fig 3, nucleotide positions 13, 54, 91, 96, 98 within the 16S rRNA V4 region are in highlighted region, which represent greater attention. Notably, position 13 is in a dim but still highlighted regions, 65 and 83 are near highlighted regions, and in fact, 83 is near two more.
(TIF)

S16 Appendix Gevers data set testing performance on different read length. To demonstrate the scalability of the Read2Pheno model to longer read lengths, we determined the sample-level classification accuracy of the Read2Pheno model trained on two different read lengths, 160 bp and 250 bp. The analysis shown here is similar to that used to generate Table 3, i.e., the obtained accuracy values are averaged and standard deviation computed over 5 experiments, in which we randomly selected training-testing data splits with replacement. The similarity in trend and values in the table demonstrate that even when trained on reads increased to 250 bp length, Read2Pheno model show stable performance, as well as increased sample-level classification accuracy. As the table showing values for 160 bp and demonstrates, our proposed method can scale to a data set with longer-length reads length data set and can produce better performance with more sequential information from longer read lengths.
(PDF)

S17 Appendix 2-D projection of embedded read vectors from *Ruminococcus* (Left) and *Blautia* (Right). Red markers represent “CD” reads, blue markers represent “Not IBD” reads. The color of a ‘×’ represents the predicted phenotype. If the predicted phenotype is the same as the true phenotype, then ‘×’s are not visible. The phenotype prediction accuracy for ***Ruminococcus*** and ***Blautia*** visualization reads are 0.84 and 0.74 respecitively.
(TIF)

S18 Appendix Average attention weights of *Enterobacter* testing sequences. Top: Average attention weights for all strains of *Enterobacter* without alignment; Bottom: Attention weights with alignment. This figure shows that our model can implicitly perform alignment to the sequence so that the attention was paid to similar position after alignment. In particular, the model learned to pay attention to important regions. Before alignment, attention are paid to a wider range of positions. However, after sequence alignment, attention weights are concentrated to a smaller range of positions (the maximum averaged attention weight is more than 13 times higher than the weight pre-alignment). This indicates that the model recognizes certain nucleotide configuration and is robust to insertions and deletions which would be accounted for by the sequence alignment.
(TIF)

S19 Appendix Secondary structure and attention weights of a *Pseudomonas aeruginosa* sequence. The attention weights (top) on a real *Pseudomonas aeruginosa* sequence provided by [68]. The positions that have an attention weight greater than the mean attention weight across the whole sequence are highlighted on the secondary structure figure (bottom).
(PDF)

S20 Appendix Mean attention weights and multiple sequence alignment results aligned for *Pseudomonas* and *Enterobacter*. (ZIP)

S21 Appendix Attention weights on variable regions per genus. The csv file contains a table for the attention weights on different variable regions per genus. The variable region indices are determined by [66] (the start and end positions for *E. Coli* in Table 1 [66]).
(CSV)

S22 Appendix Attention weights visualization of a body site classification model trained by reads from 400 AGP samples. This figure compares the average *Prevotella* reads attention and nucleotide frequency entropy in form of nucleotide sequence logo. A: oral reads; B: fecal reads; C: skin reads; D: overall attention. The *Prevotella* visualization reads are classified to the correct body site source with 68.46% accuracy (the balance accuracy is 66.45%).
(TIF)

S23 Appendix Attention weights visualization of a body site classification model trained by reads from 750 AGP samples. This figure compares the average *Prevotella* reads attention and nucleotide frequency entropy in form of nucleotide sequence logo. A: oral reads; B: fecal reads; C: skin reads; D: overall attention. The *Prevotella* visualization reads are classified to the correct body site source with 91.49% accuracy (the balance accuracy is 67.86%).
(TIF)

S24 Appendix Attention weights visualization of a Crohn’s disease classification model trained by reads from 160 Gevers samples. This figure compares the average *Blautia* reads attention and nucleotide frequency entropy in form of nucleotide sequence logo. A: “CD” reads; B: “Not IBD” reads; C: overall attention. The *Blautia* visualization reads are classified to the correct host health status with 6.49% accuracy (the balance accuracy is 22.41% due to high false negative rate on this genus).
(TIF)

S25 Appendix Attention weights visualization of a Crohn’s disease classification model trained by reads from 400 Gevers samples. This figure compares the average *Blautia* reads attention and nucleotide frequency entropy in form of nucleotide sequence logo. A: “CD” reads; B: “Not IBD” reads; C: overall attention. The *Blautia* visualization reads are classified to the correct host health status with 75.34% accuracy (the balance accuracy is 48.93%).
(TIF)

## References

[1] Navas-Molina JA, Hyde ER, Sanders JG, Knight R. The microbiome and big data. Current Opinion in Systems Biology. 2017;4:92–96. 10.1016/j.coisb.2017.07.003 PMC10019530 36937228

[2] Bernhard A, Colbert D, McManus J, Field K. Microbial community dynamics based on 16S rRNA gene profiles in a Pacific Northwest estuary and its tributaries. FEMS microbiology ecology. 2005;52 1:115–28. doi: 10.1016/j.femsec.2004.10.016 16329898

[3] Nakatsu CH, Byappanahalli MN, Nevers MB. Bacterial Community 16S rRNA Gene Sequencing Characterizes Riverine Microbial Impact on Lake Michigan. Frontiers in Microbiology. 2019;10. doi: 10.3389/fmicb.2019.00996 PMC6527805 31139161

[4] Ross EM, Moate PJ, Marett LC, Cocks BG, Hayes BJ. Metagenomic Predictions: From Microbiome to Complex Health and Environmental Phenotypes in Humans and Cattle. PLOS ONE. 2013;8(9):1–8. doi: 10.1371/journal.pone.0073056 PMC3762846 24023808

[5] Gevers D, Kugathasan S, Denson L, Vázquez-Baeza Y, Treuren WWV, Ren B, et al. The Treatment-Naïve Microbiome in New-Onset Crohn’s Disease. Cell host & microbe. 2014;15 3:382–392. doi: 10.1016/j.chom.2014.02.005 24629344 PMC4059512

[6] Carrieri AP, Haiminen N, Parida L. Host Phenotype Prediction from Differentially Abundant Microbes Using RoDEO. In: CIBB; 2016.

[7] Asgari E, Garakani K, McHardy AC, Mofrad MRK. MicroPheno: predicting environments and host phenotypes from 16S rRNA gene sequencing using a k-mer based representation of shallow sub-samples. Bioinformatics. 2018;34(13):i32–i42. doi: 10.1093/bioinformatics/bty296 29950008 PMC6022683

[8] Navarro G, Sharma A, Dugas LR, Forrester T, Gilbert JA, Layden BT. Gut microbial features can predict host phenotype response to protein deficiency. Physiological Reports. 2018;6(23):e13932. doi: 10.14814/phy2.13932 30516001 PMC6280014

[9] Hunter P. Extended phenotype redux. How far can the reach of genes extend in manipulating the environment of an organism? EMBO reports. 2009;10 3:212–5. 19255576 10.1038/embor.2009.18 PMC2658563

[10] Cullen CM, Aneja KK, Beyhan S, Cho CE, Woloszynek S, Convertino M, et al. Emerging Priorities for Microbiome Research. Frontiers in Microbiology. 2020;11. doi: 10.3389/fmicb.2020.00136 32140140 PMC7042322

[11] Fischbach M. Microbiome: Focus on Causation and Mechanism. Cell. 2018;174:785–790. doi: 10.1016/j.cell.2018.07.038 30096310 PMC6094951

[12] Ward TL, Larson J, Meulemans J, Hillmann B, Lynch J, Sidiropoulos D, et al. BugBase predicts organism-level microbiome phenotypes. bioRxiv. 2017;.

[13] Lu K, Mahbub R, Cable P, Ru H, Parry N, Bodnar W, et al. Gut Microbiome Phenotypes Driven by Host Genetics Affect Arsenic Metabolism. Chemical Research in Toxicology. 2014;27:172–174. doi: 10.1021/tx400454z 24490651 PMC3997221

[14] Stanislawski M, Dabelea D, Lange LA, Wagner B, Lozupone C. Gut microbiota phenotypes of obesity. NPJ Biofilms and Microbiomes. 2019;5. doi: 10.1038/s41522-019-0091-8 PMC6603011 31285833

[15] Lynch JB, Hsiao EY. Microbiomes as sources of emergent host phenotypes. Science. 2019;365:1405–1409. doi: 10.1126/science.aay0240 31604267

[16] Ross E, Moate P, Marett L, Cocks B, Hayes B. Metagenomic Predictions: From Microbiome to Complex Health and Environmental Phenotypes in Humans and Cattle. PLoS ONE. 2013;8. doi: 10.1371/journal.pone.0073056 PMC3762846 24023808

[17] Bhattacharjee A, Veličković D, Wietsma T, Bell SL, Jansson J, Hofmockel K, et al. Visualizing Microbial Community Dynamics via a Controllable Soil Environment. mSystems. 2020;5. doi: 10.1128/mSystems.00645-19 32047062 PMC7018529

[18] Nishiyama E, Higashi K, Mori H, Suda K, Nakamura H, Omori S, et al. The Relationship Between Microbial Community Structures and Environmental Parameters Revealed by Metagenomic Analysis of Hot Spring Water in the Kirishima Area, Japan. Frontiers in Bioengineering and Biotechnology. 2018;6. doi: 10.3389/fbioe.2018.00202 30619848 PMC6306410

[19] Pedron R, Esposito A, Bianconi I, Pasolli E, Tett A, Asnicar F, et al. Genomic and metagenomic insights into the microbial community of a thermal spring. Microbiome. 2019;7. doi: 10.1186/s40168-019-0625-6 30674352 PMC6343286

[20] Pollock J, Glendinning L, Wisedchanwet T, Watson M. The Madness of Microbiome: Attempting To Find Consensus “Best Practice” for 16S Microbiome Studies. Applied and Environmental Microbiology. 2018;84. doi: 10.1128/AEM.02627-17 PMC5861821 29427429

[21] Statnikov A, Henaff M, Narendra V, Konganti K, Li Z, Yang L, et al. A comprehensive evaluation of multicategory classification methods for microbiomic data. Microbiome. 2013;1(1):11. doi: 10.1186/2049-2618-1-11 24456583 PMC3960509

[22] Simonyan K, Vedaldi A, Zisserman A. Deep Inside Convolutional Networks: Visualising Image Classification Models and Saliency Maps. CoRR. 2013;abs/1312.6034.

[23] Shrikumar A, Greenside P, Shcherbina A, Kundaje A. Not Just a Black Box: Learning Important Features Through Propagating Activation Differences. ArXiv. 2016;.

[24] Simonyan K, Vedaldi A, Zisserman A. Deep Inside Convolutional Networks: Visualising Image Classification Models and Saliency Maps. ArXiv. 2013; p. 1–8.

[25] Yosinski J, Clune J, Nguyen A, Fuchs T, Lipson H. Understanding Neural Networks Through Deep Visualization. ArXiv. 2015;.

[26] Min S, Lee B, Yoon S. Deep learning in bioinformatics. Briefings in Bioinformatics. 2016; p. bbw068. doi: 10.1093/bib/bbw068 27473064

[27] Ditzler G, Polikar R, Rosen G. Multi-Layer and Recursive Neural Networks for Metagenomic Classification. IEEE Transactions on NanoBioscience. 2015;14:608–616. doi: 10.1109/TNB.2015.2461219 26316190

[28] Lanchantin J, Singh R, Lin Z, Qi Y. Deep Motif: Visualizing Genomic Sequence Classifications. ArXiv. 2016;.

[29] Deming L, Targ S, Sauder N, Almeida D, Ye CJ. Genetic Architect: Discovering Genomic Structure with Learned Neural Architectures. ArXiv. 2016;.

[30] Poplin R, Newburger D, Dijamco J, Nguyen NN, Loy D, Gross S, et al. Creating a universal SNP and small indel variant caller with deep neural networks. bioRxiv. 2017;. 10.1038/nbt.4235 30247488

[31] Hess M, Lenz S, Blätte TJ, Bullinger L, Binder H. Partitioned learning of deep Boltzmann machines for SNP data. Bioinformatics. 2017;33(20):3173–3180. doi: 10.1093/bioinformatics/btx408 28655145

[32] Busia A, Dahl GE, Fannjiang C, Alexander DH, Dorfman E, Poplin R, et al. A deep learning approach to pattern recognition for short DNA sequences. bioRxiv. 2018.

[33] Quang D, Xie X. DanQ: A hybrid convolutional and recurrent deep neural network for quantifying the function of DNA sequences. Nucleic Acids Research. 2016;44:gkw226. doi: 10.1093/nar/gkw226 PMC4914104 27084946

[34] Chen Y, Li Y, Narayan R, Subramanian A, Xie X. Gene expression inference with deep learning. Bioinformatics. 2016;32(12):1832–1839. doi: 10.1093/bioinformatics/btw074 26873929 PMC4908320

[35] Lo C, Marculescu R. MetaNN: accurate classification of host phenotypes from metagenomic data using neural networks. BMC Bioinformatics. 2019;20. doi: 10.1186/s12859-019-2833-2 PMC6584521 31216991

[36] Nguyen TH, Prifti E, Chevaleyre Y, Sokolovska N, Zucker JD. Disease Classification in Metagenomics with 2D Embeddings and Deep Learning. ArXiv. 2018;abs/1806.09046.

[37] Reiman D, Metwally A, Sun J, Dai Y. PopPhy-CNN: A Phylogenetic Tree Embedded Architecture for Convolutional Neural Networks to Predict Host Phenotype from Metagenomic Data. IEEE Journal of Biomedical and Health Informatics. 2020; p. 1–1. 10.1109/JBHI.2020.2993761 32396115

[38] Woloszynek S, Zhao Z, Chen J, Rosen GL. 16S rRNA sequence embeddings: Meaningful numeric feature representations of nucleotide sequences that are convenient for downstream analyses. PLOS Computational Biology. 2019;15(2):1–25. doi: 10.1371/journal.pcbi.1006721 PMC6407789 30807567

[39] Lapierre N, Ju C, Zhou G, Wang W. MetaPheno: A critical evaluation of deep learning and machine learning in metagenome-based disease prediction. Methods. 2019;. doi: 10.1016/j.ymeth.2019.03.003 30885720 PMC6708502

[40] Murdoch WJ, Singh C, Kumbier K, Abbasi-Asl R, Yu B. Definitions, methods, and applications in interpretable machine learning. Proceedings of the National Academy of Sciences. 2019;116:22071–22080. doi: 10.1073/pnas.1900654116 PMC6825274 31619572

[41] Eren AM, Maignien L, Sul WJ, Murphy LG, Grim SL, Morrison HG, et al. Oligotyping: differentiating between closely related microbial taxa using 16S rRNA gene data. Methods in Ecology and Evolution. 2013;4(12):1111–1119. doi: 10.1111/2041-210X.12114 24358444 PMC3864673

[42] Aun E, Brauer A, Kisand V, Tenson T, Remm M. A k-mer-based method for the identification of phenotype-associated genomic biomarkers and predicting phenotypes of sequenced bacteria. PLOS Computational Biology. 2018;14(10):1–17. doi: 10.1371/journal.pcbi.1006434 PMC6211763 30346947

[43] Alipanahi B, Delong A, Weirauch MT, Frey BJ. Predicting the sequence specificities of DNA- and RNA-binding proteins by deep learning. Nature Biotechnology. 2015;33:831–838. doi: 10.1038/nbt.3300 26213851

[44] Lanchantin J, Singh R, Wang B, Qi Y. Deep Motif Dashboard: Visualizing and Understanding Genomic Sequences Using Deep Neural Networks. ArXiv. 2016;. 10.1142/9789813207813_0025 PMC5787355 27896980

[45] Bahdanau D, Cho K, Bengio Y. Neural Machine Translation by Jointly Learning to Align and Translate. CoRR. 2015;abs/1409.0473.

[46] Yang Z, Yang D, Dyer C, He X, Smola AJ, et al. Hierarchical Attention Networks for Document Classification. In: HLT-NAACL; 2016.

[47] Zhou P, Shi W, Tian J, Qi Z, Li B, Hao H, et al. Attention-Based Bidirectional Long Short-Term Memory Networks for Relation Classification. In: ACL; 2016.

[48] Liu Q, Zhang H, Zeng Y, Huang Z, Wu Z. Content Attention Model for Aspect Based Sentiment Analysis. In: Proceedings of the 2018 World Wide Web Conference. WWW’18. Republic and Canton of Geneva, Switzerland: International World Wide Web Conferences Steering Committee; 2018. p. 1023–1032. Available from: 10.1145/3178876.3186001.

[49] McDonald D, Hyde ER, Debelius JW, Morton JT, González A, Ackermann G, et al. American Gut: an Open Platform for Citizen Science Microbiome Research. mSystems. 2018;3. doi: 10.1128/mSystems.00031-18 29795809 PMC5954204

[50] Quast C, Pruesse E, Yilmaz P, Gerken J, Schweer T, Yarza P, et al. The SILVA ribosomal RNA gene database project: improved data processing and web-based tools. Nucleic Acids Research. 2012;41(D1):D590–D596. doi: 10.1093/nar/gks1219 23193283 PMC3531112

[51] Yilmaz P, Parfrey LW, Yarza P, Gerken J, Pruesse E, Quast C, et al. The SILVA and “All-species Living Tree Project (LTP)” taxonomic frameworks. Nucleic Acids Research. 2013;42(D1):D643–D648. doi: 10.1093/nar/gkt1209 24293649 PMC3965112

[52] Raffel C, Ellis DPW. Feed-Forward Networks with Attention Can Solve Some Long-Term Memory Problems. ArXiv. 2015;abs/1512.08756.

[53] Callahan BJ, McMurdie PJ, Rosen MJ, Han AW, Johnson AJA, Holmes SP. DADA2: High resolution sample inference from Illumina amplicon data. Nature methods. 2016;13:581–583. doi: 10.1038/nmeth.3869 27214047 PMC4927377

[54] Gregory Caporaso J, Kuczynski J, Stombaugh J, Bittinger K, Bushman FD, Costello EK, et al. QIIME allows analysis of high-throughput community sequencing data. Nat Met 7: 335-336. Nature methods. 2010;7:335–6. doi: 10.1038/nmeth.f.303 20383131 PMC3156573

[55] Cole JR, Wang Q, Fish JA, Chai B, McGarrell DM, Sun Y, et al. Ribosomal Database Project: data and tools for high throughput rRNA analysis. Nucleic Acids Research. 2013;42(D1):D633–D642. doi: 10.1093/nar/gkt1244 24288368 PMC3965039

[56] Towns J, Cockerill T, Dahan M, Foster I, Gaither K, Grimshaw A, et al. XSEDE: Accelerating Scientific Discovery. Computing in Science Engineering. 2014;16(5):62–74. doi: 10.1109/MCSE.2014.80

[57] Crooks GE, Hon G, Chandonia JM, Brenner SE. WebLogo: a Sequence Logo Generator. Genome research. 2004;14:1188–90. doi: 10.1101/gr.849004 15173120 PMC419797

[58] ImportanceOfBeingErnest. sequence logos in matplotlib: aligning xticks; 2017. Available from: https://stackoverflow.com/a/42631740.

[59] Fu L, Niu B, Zhu Z, Wu S, Li W. CD-HIT: accelerated for clustering the next-generation sequencing data. Bioinformatics. 2012;28:3150–3152. doi: 10.1093/bioinformatics/bts565 23060610 PMC3516142

[60] Rosenberg A, Hirschberg J. V-Measure: A Conditional Entropy-Based External Cluster Evaluation Measure. In: EMNLP-CoNLL; 2007.

[61] Hubert L, Arabie P. Comparing partitions. Journal of Classification. 1985;2:193–218. doi: 10.1007/BF01908075

[62] Duvallet C, Gibbons SM, Gurry T, Irizarry RA, Alm EJ. Meta-analysis of gut microbiome studies identifies disease-specific and shared responses. Nature Communications. 2017;8(1):1784. doi: 10.1038/s41467-017-01973-8 PMC5716994 29209090

[63] Wagner J, Catto-Smith AG, Cameron DJS, Kirkwood CD. Pseudomonas Infection in Children with Early-onset Crohn’s Disease: An Association with a Mutation Close to PSMG1. Inflammatory Bowel Diseases. 2012;19(4):E58–E59. doi: 10.1002/ibd.23017 22593026

[64] De Cruz P, Prideaux L, Wagner J, Ng SC, McSweeney C, Kirkwood C, et al. Characterization of the gastrointestinal microbiota in health and inflammatory bowel disease. Inflammatory Bowel Diseases. 2012;18(2):372–390. doi: 10.1002/ibd.21751 21604329

[65] Wagner J, Short K, Catto-Smith AG, Cameron DJS, Bishop RF, Kirkwood CD. Identification and Characterisation of Pseudomonas 16S Ribosomal DNA from Ileal Biopsies of Children with Crohn’s Disease. PLOS ONE. 2008;3(10):1–7. doi: 10.1371/journal.pone.0003578 PMC2572839 18974839

[66] Yang B, Wang Y, Qian PY. Sensitivity and correlation of hypervariable regions in 16S rRNA genes in phylogenetic analysis. BMC Bioinformatics. 2016;17. doi: 10.1186/s12859-016-0992-y PMC4802574 27000765

[67] Miller MA, Pfeiffer W, Schwartz T. Creating the CIPRES Science Gateway for inference of large phylogenetic trees. In: 2010 Gateway Computing Environments Workshop (GCE); 2010. p. 1–8.

[68] Cannone JJ, Subramanian S, Schnare MN, Collett JR, D’Souza LM, Du Y, et al. The Comparative RNA Web (CRW) Site: an online database of comparative sequence and structure information for ribosomal, intron, and other RNAs. BMC Bioinformatics. 2002;3(1):2. doi: 10.1186/1471-2105-3-2 11869452 PMC65690

[69] Vinje H, Almøy T, Liland K, Snipen L. A systematic search for discriminating sites in the 16S ribosomal RNA gene. Microbial informatics and experimentation. 2014;4:2. doi: 10.1186/2042-5783-4-2 24467869 PMC3910680

[70] Mallick H, Franzosa E, Mclver L, Banerjee S, Sirota-Madi A, Kostic A, et al. Predictive metabolomic profiling of microbial communities using amplicon or metagenomic sequences. Nature Communications. 2019;10:3136–3146. doi: 10.1038/s41467-019-10927-1 31316056 PMC6637180

[71] Graspeuntner S, Loeper N, Künzel S, Baines J, Rupp J. Selection of validated hypervariable regions is crucial in 16S-based microbiota studies of the female genital tract. Scientific Reports. 2018;8. doi: 10.1038/s41598-018-27757-8 PMC6018735 29946153

[72] Chen Z, Hui PC, Hui M, Yeoh YK, Wong PY, Chan MCW, et al. Impact of Preservation Method and 16S rRNA Hypervariable Region on Gut Microbiota Profiling. mSystems. 2019;4(1). doi: 10.1128/mSystems.00271-18 30834331 PMC6392095

[73] McDonald D, Birmingham A, Knight R. Context and the human microbiome. Microbiome. 2015;3(1):52. doi: 10.1186/s40168-015-0117-2 26530830 PMC4632476

[74] Lozupone C, Knight R. UniFrac: a New Phylogenetic Method for Comparing Microbial Communities. Applied and Environmental Microbiology. 2005;71(12):8228–8235. doi: 10.1128/AEM.71.12.8228-8235.2005 16332807 PMC1317376

[75] McDonald D, Xu Z, Hyde ER, Knight R. Ribosomal RNA, the lens into life. Cold Spring Harbor Laboratory Press for the RNA Society. 2015. 10.1261/rna.050799.115 PMC4371336 25780194

[76] Ha F, Khalil H. Crohn’s disease: a clinical update. Therapeutic Advances in Gastroenterology. 2015;8(6):352–359. doi: 10.1177/1756283X15592585 26557891 PMC4622286

[77] Pascal V, Pozuelo M, Borruel N, Casellas F, Campos D, Santiago A, et al. A microbial signature for Crohn’s disease. Gut. 2017;66(5):813–822. doi: 10.1136/gutjnl-2016-313235 28179361 PMC5531220

[78] Bishop CM. Pattern Recognition and Machine Learning (Information Science and Statistics); 2006.

[79] Zhang C, Bengio S, Hardt M, Recht B, Vinyals O. Understanding deep learning requires rethinking generalization. ArXiv. 2017;abs/1611.03530.

[80] McIntyre ABR, Ounit R, Afshinnekoo E, Prill RJ, Hénaff E, Alexander N, et al. Comprehensive benchmarking and ensemble approaches for metagenomic classifiers. Genome Biology. 2017;18(1):182. doi: 10.1186/s13059-017-1299-7 28934964 PMC5609029

[81] Meyer F, Fritz A, Deng ZL, Koslicki D, Gurevich A, Robertson G, et al. Critical Assessment of Metagenome Interpretation—the second round of challenges. bioRxiv. 2021. 10.1038/s41592-022-01431-4 PMC9007738 35396482

[82] Johnson JS, Spakowicz DJ, young Hong B, Petersen LM, Demkowicz P, Chen L, et al. Evaluation of 16S rRNA gene sequencing for species and strain-level microbiome analysis. Nature Communications. 2019;10. doi: 10.1038/s41467-019-13036-1 31695033 PMC6834636

[83] Willis C, Desai DK, Laroche J. Influence of 16S rRNA variable region on perceived diversity of marine microbial communities of the Northern North Atlantic. FEMS Microbiology Letters. 2019;366. doi: 10.1093/femsle/fnz152 PMC6673769 31344223

[84] Wang Q, Garrity G, Tiedje J, Cole J. Naïve Bayesian Classifier for Rapid Assignment of rRNA Sequences into the New Bacterial Taxonomy. Applied and Environmental Microbiology. 2007;73:5261–5267. doi: 10.1128/AEM.00062-07 17586664 PMC1950982

