# Supplementary figures and images for: Learning, visualizing and exploring 16S rRNA structure using an attention-based deep neural network
Source: PLoS Comput Biol. 2021 Sep 22;17(9):e1009345. doi: 10.1371/journal.pcbi.1009345 (PMC8496832; doi:10.1371/journal.pcbi.1009345)

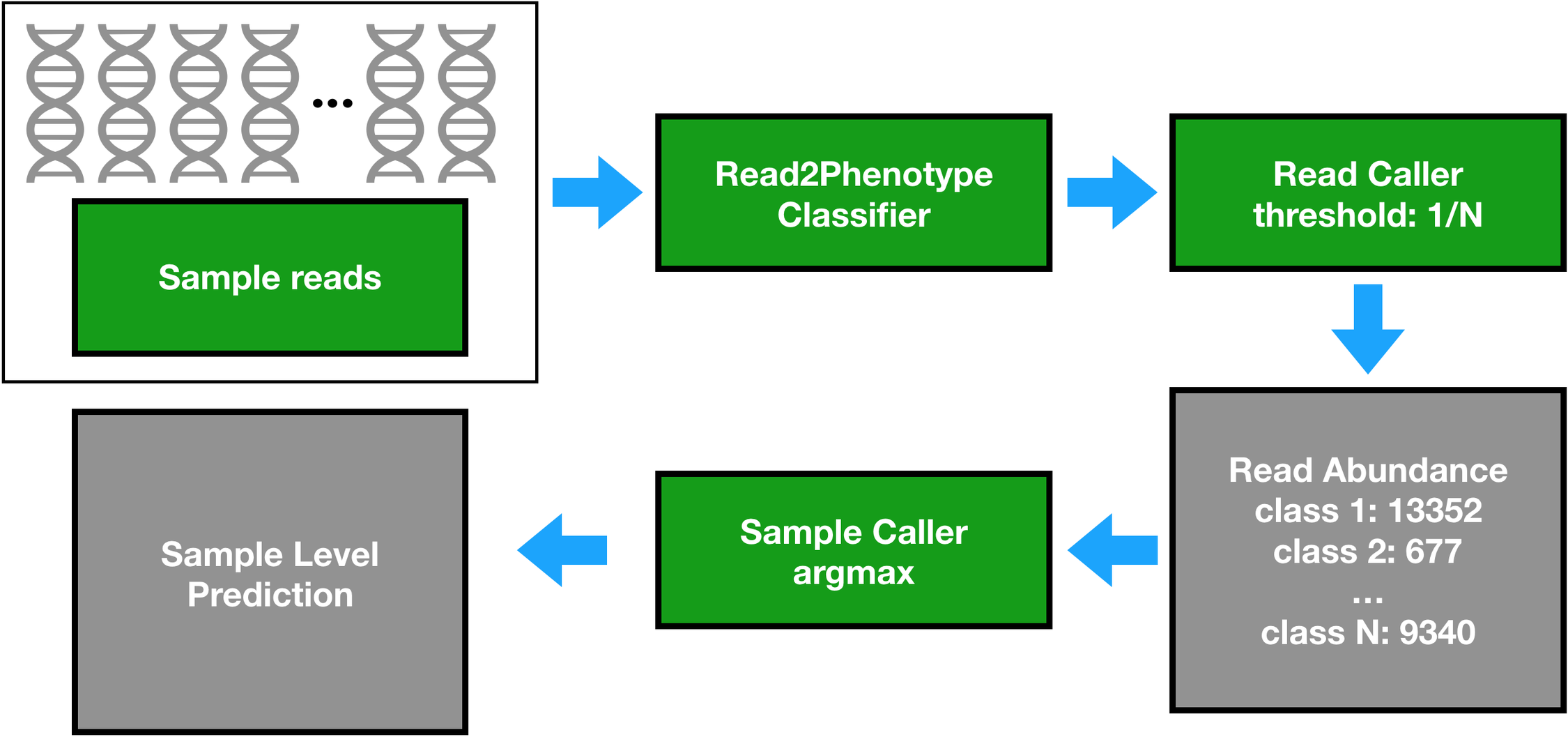

Supplement: S2 Appendix — The read caller threshold is 1N where N is the number of classes. (TIF) [file pcbi.1009345.s002.tif]

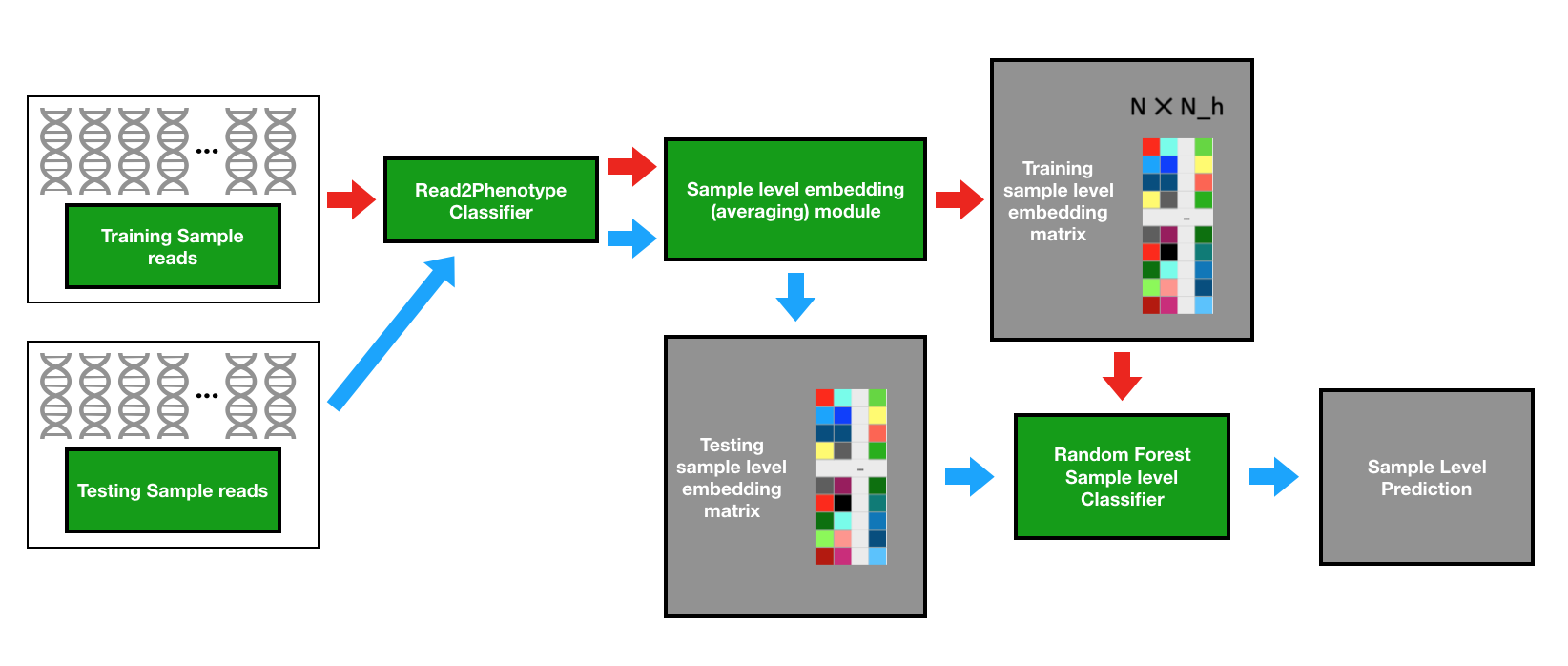

Supplement: S3 Appendix — Sample-level embedding is calculated by averaging all the read-level embedding vectors per sample. Then, a random forest classifier is trained based on the sample embedding matrix (an N by Nh matrix where N is the total number of samples in training set and Nh is the number of hidden nodes in Bi-LSTM layer). Once the sample-level random forest classifier is trained, this model can be used to perform sample-level classification by taking the sample embedding vectors as input. The training and testing process are labeled by red and blue arrows correspondingly. (TIF) [file pcbi.1009345.s003.tif]

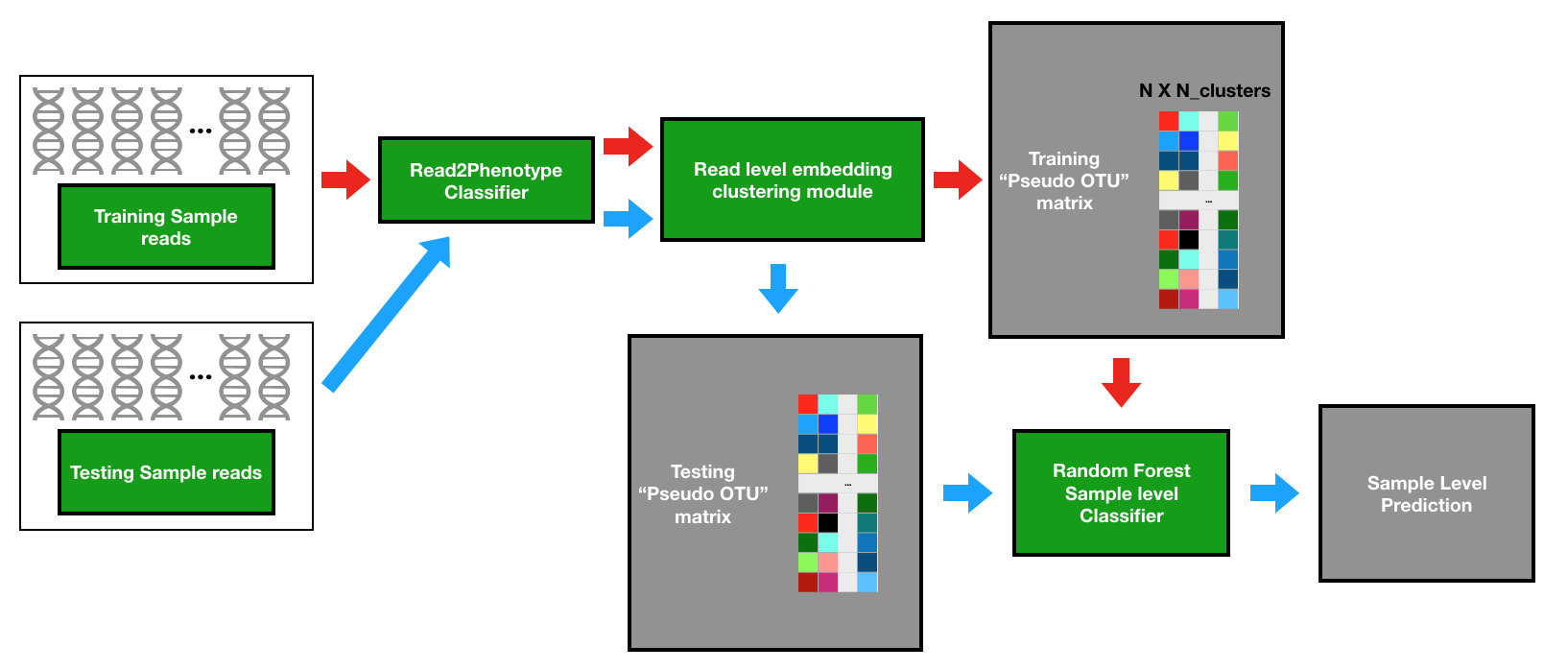

Supplement: S4 Appendix — Training reads are clustered into Nclusters = 1000 clusters (Pseudo OTUs) by the read-level embedding clustering module using a k − means algorithm. Then, all training reads per sample are mapped to the closest Pseudo OTUs to form Pseudo OTUs abundance table. Similar to sample-level embedding method, a random forest classifier can be trained to perform sample-level prediction using such Pseudo OTUs table (The training and testing process are labeled by red and blue arrows correspondingly). (TIF) [file pcbi.1009345.s004.tif]

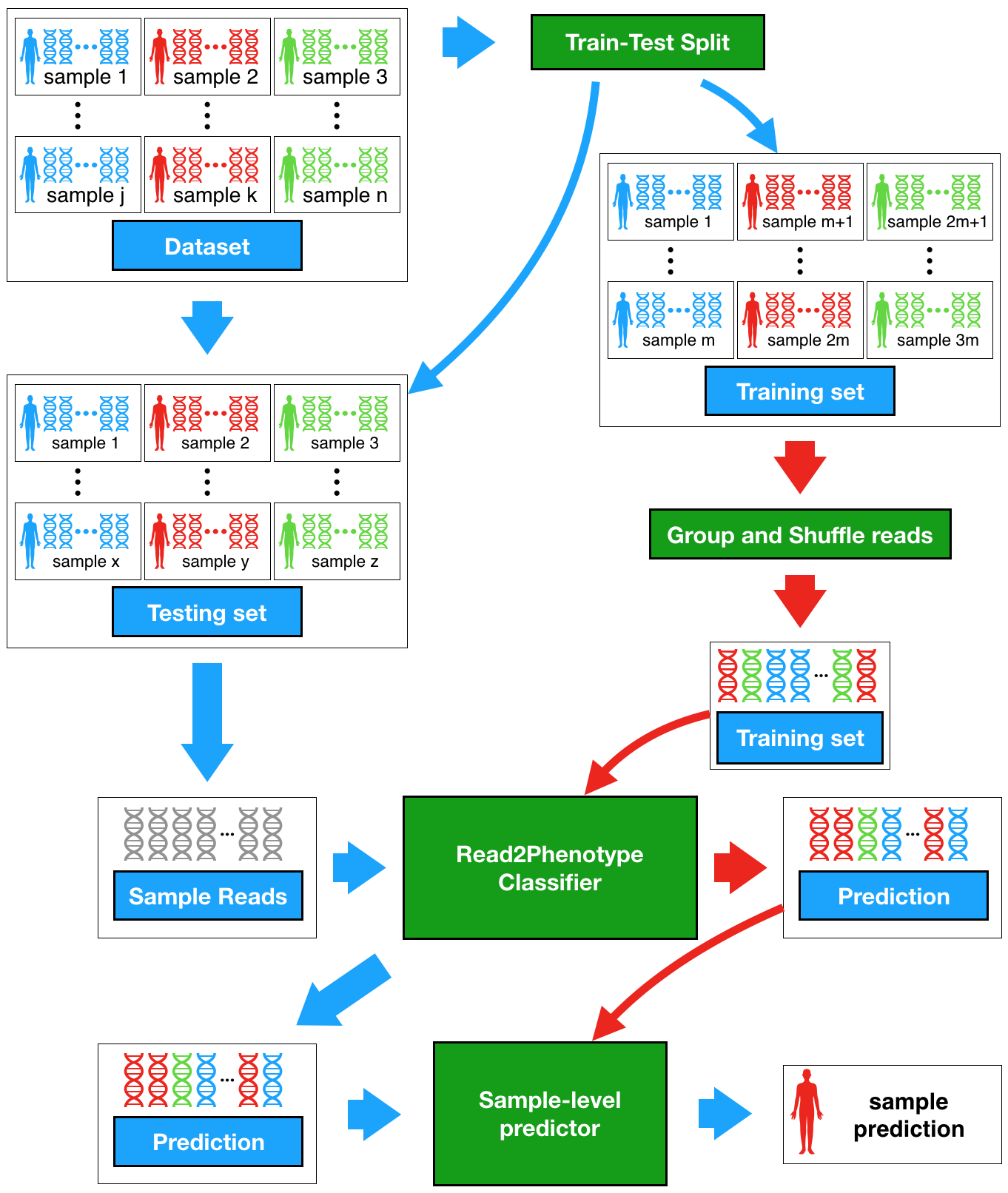

Supplement: S6 Appendix — Samples are split into train and test set. Training set is used to train a Read2Pheno classifier and a sample-level predictor. The testing set is used to evaluate the performance. (TIF) [file pcbi.1009345.s006.tif]

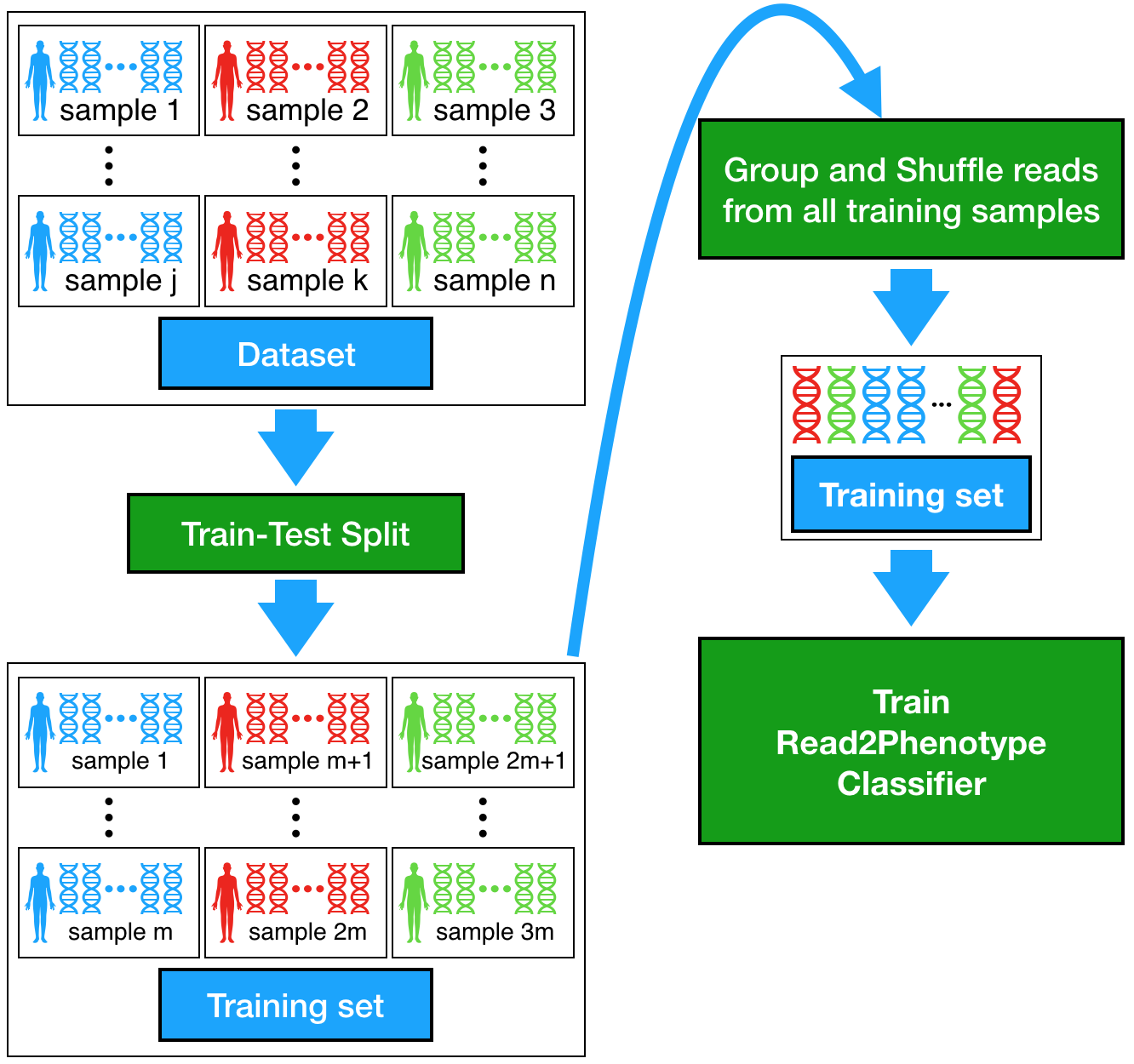

Supplement: S7 Appendix — All reads in the training set are labeled by the sample-level label (the body site the original sample was collected from). Then the reads are grouped together and shuffled for training the Read2Pheno classifier. (TIF) [file pcbi.1009345.s007.tif]

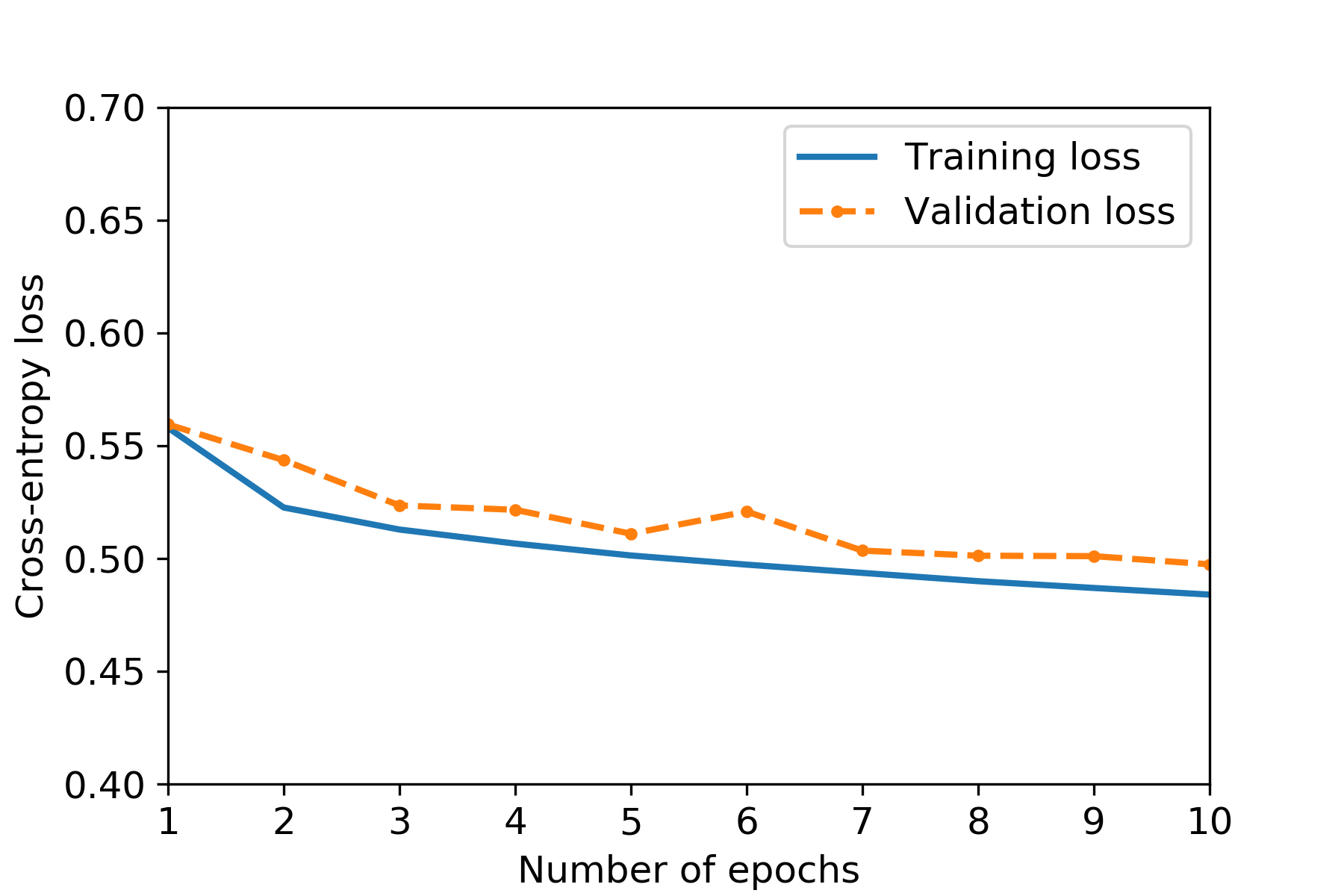

Supplement: S9 Appendix — This figure shows the cross-entropy loss of our model in training and validation as a function of epochs. From this figure, we can see that the validation loss stabilized after epoch number 7. (TIF) [file pcbi.1009345.s009.tif]

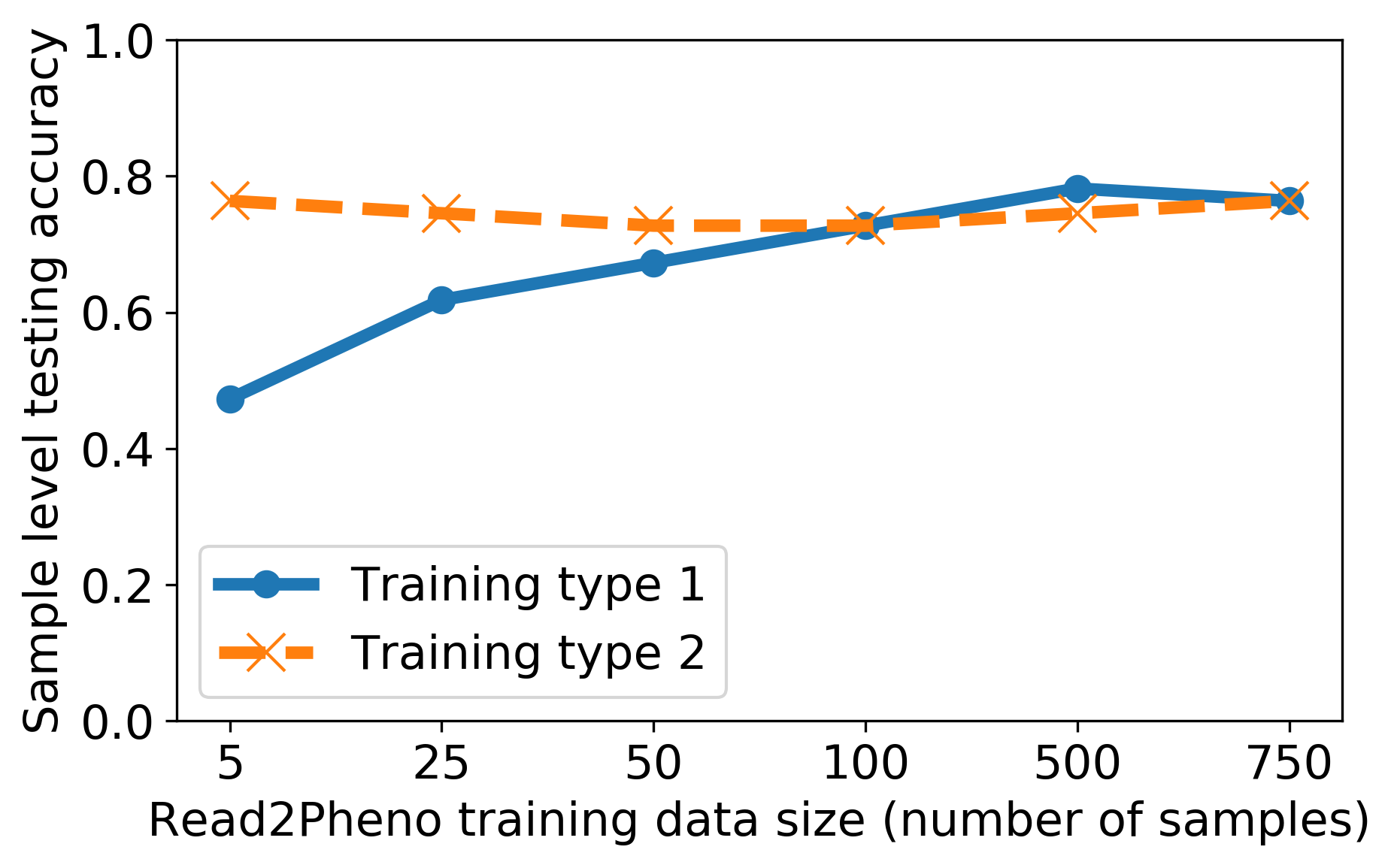

Supplement: S10 Appendix — To evaluate the influence of the training size for Read2Pheno classifier training, we designed an independent experiment: we first hold out 55 samples for testing, then we train the Read2Pheno model with reads from 5, 25, 50, 100, 500 and 750 samples from the rest of samples and evaluate the sample-level performance of those models by the 55 samples in the held-out testing set. (Here, we use the sample-level embedding method for sample prediction). To determine how much sample-level prediction quality depends on the size of the Read2Pheno training set as compared to the size of the downstream sample-level training (i.e. Random Forest) set, we measure test set prediction accuracy where sample-level Random Forest is trained 1) on the same training set as the Read2Pheno classifier, and 2) on all 750 samples irrespective of the number of samples used to train Read2Pheno. The blue curve shows the training type 1 and the orange line shows the training type 2. The blue curve shows that as more samples used for training, the sample-level accuracy increases. The orange curve shows that although Read2Pheno classifiers are trained with different samples, as long as the sample-level prediction model is trained with more samples, the performance is generally stable. (TIF) [file pcbi.1009345.s010.tif]

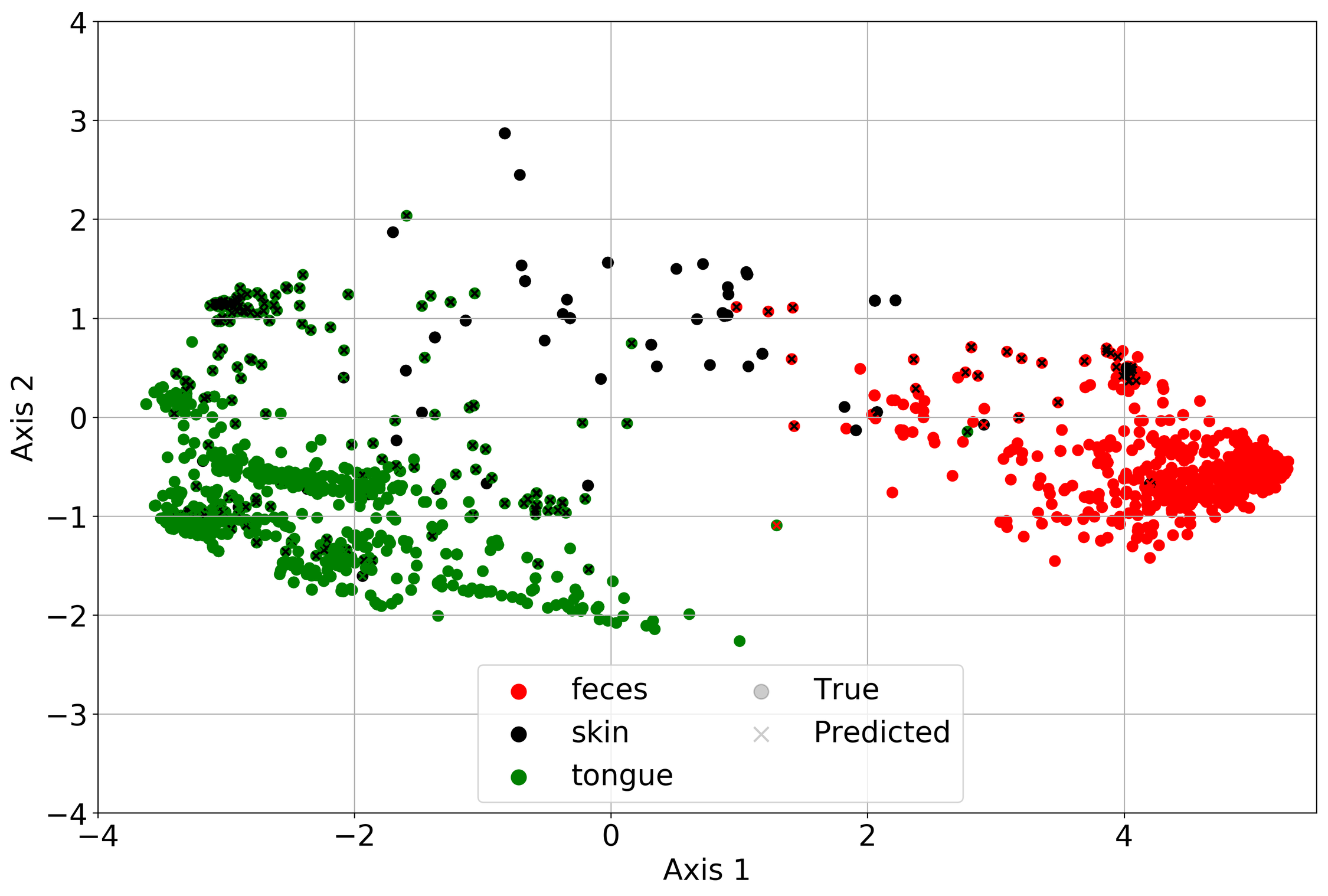

Supplement: S12 Appendix — Red markers represent fecal reads, green markers represent oral reads and black markers represent skin reads. The color of a ‘×’ represents the predicted body site. If the predicted body site is the same as the true body site, then ‘×’s are not visible. The body site prediction accuracy for Prevotella visualization reads is 0.9131. (TIF) [file pcbi.1009345.s012.tif]

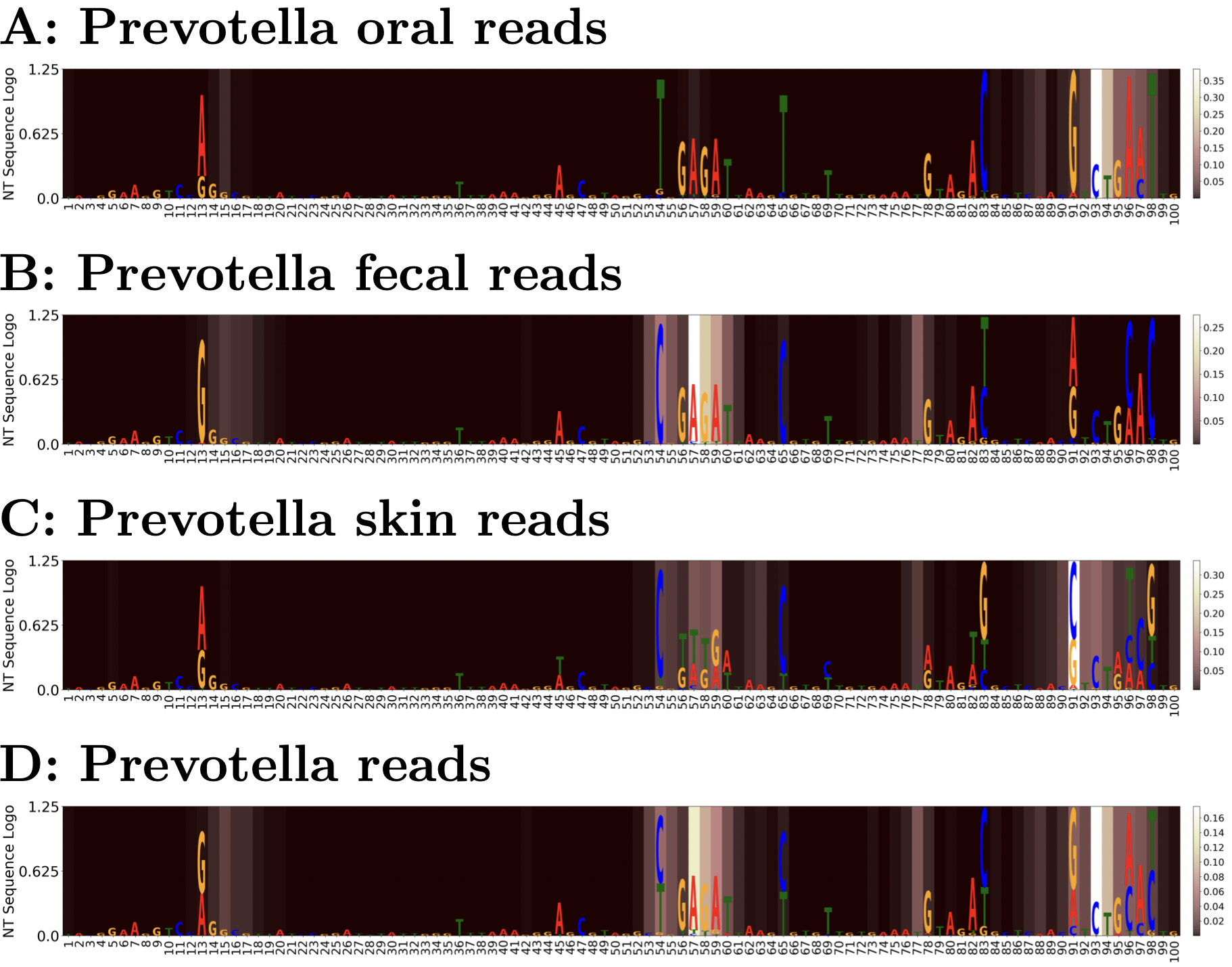

Supplement: S13 Appendix — Comparison between average Prevotella reads attention weights and nucleotide frequency entropy in form of nucleotide sequence logo. A: oral reads; B: fecal reads; C: skin reads; D: overall attention. In each body site, nucleotide frequencies are scaled by the overall entropy for all Prevotella testing reads and plotted as a sequence logo, with average attention weights represented by a color map where lighter background shading represents larger values for attention weights, in contrast with darker background shading for smaller attention weights. Attention weights are not smoothed by the moving average of window size of 9 as opposed to Fig 3. (TIF) [file pcbi.1009345.s013.tif]

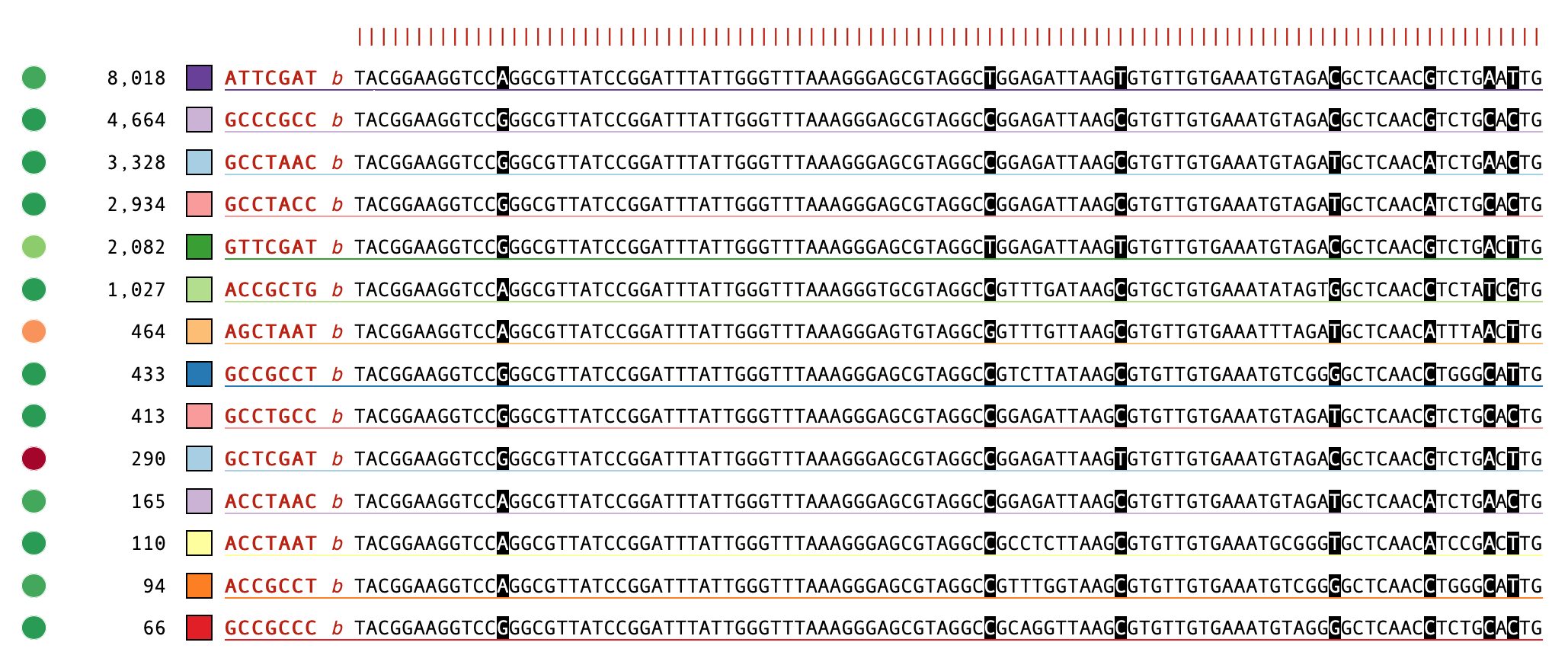

Supplement: S14 Appendix — Top 7 Oligotypes found in Prevotella reads. The number on the left hand side of the figure shows the number of reads having a certain type of Oligotyping patterns (the nucleotide combination in black positions). (TIF) [file pcbi.1009345.s014.tif]

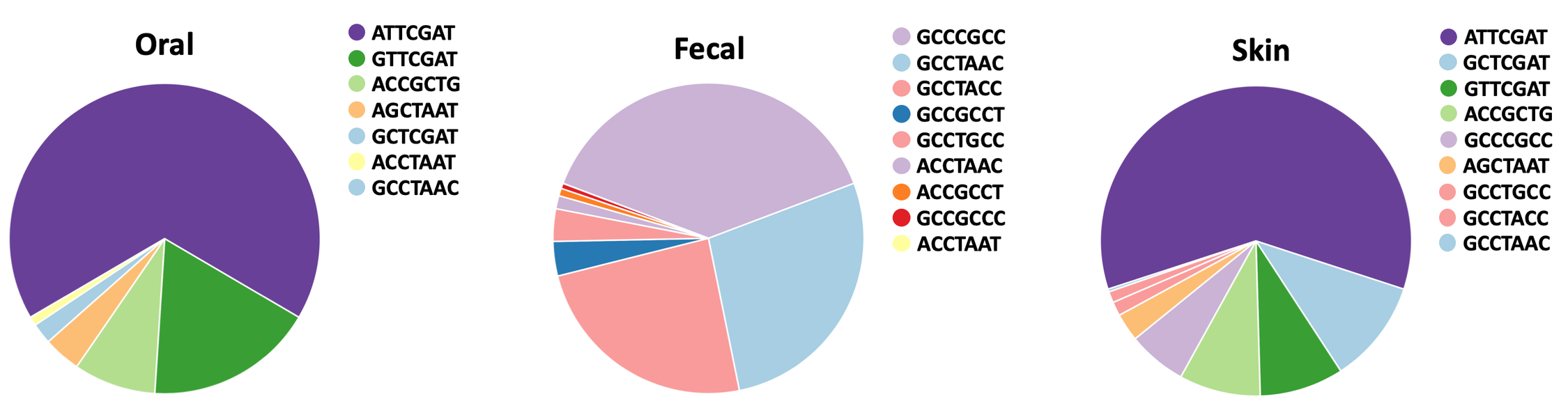

Supplement: S15 Appendix — Different 7-oligotypes configurations can be found in different body sites. Fecal samples have a distinctive configuration as compared with oral and skin samples. Of note, Oligotyping for 7 positions picks positions 13, 54, 65, 83, 91, 96, and 98. In our model, shown in Fig 3, nucleotide positions 13, 54, 91, 96, 98 within the 16S rRNA V4 region are in highlighted region, which represent greater attention. Notably, position 13 is in a dim but still highlighted regions, 65 and 83 are near highlighted regions, and in fact, 83 is near two more. (TIF) [file pcbi.1009345.s015.tif]

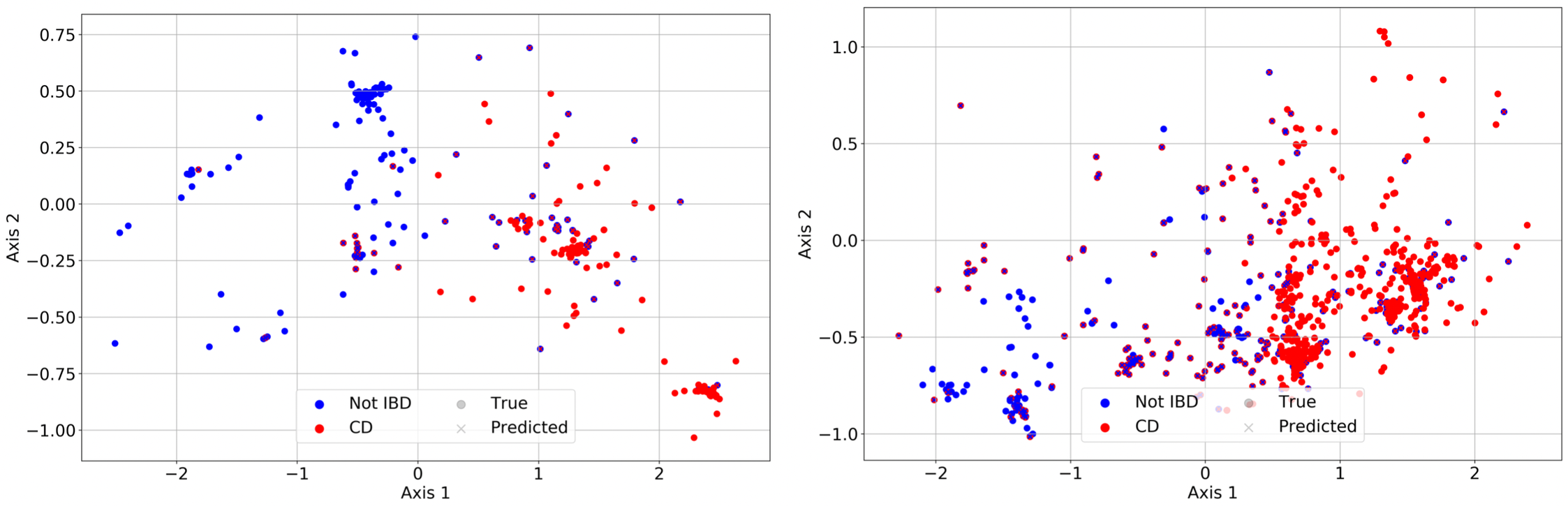

Supplement: S17 Appendix — Red markers represent “CD” reads, blue markers represent “Not IBD” reads. The color of a ‘×’ represents the predicted phenotype. If the predicted phenotype is the same as the true phenotype, then ‘×’s are not visible. The phenotype prediction accuracy for Ruminococcus and Blautia visualization reads are 0.84 and 0.74 respecitively. (TIF) [file pcbi.1009345.s017.tif]

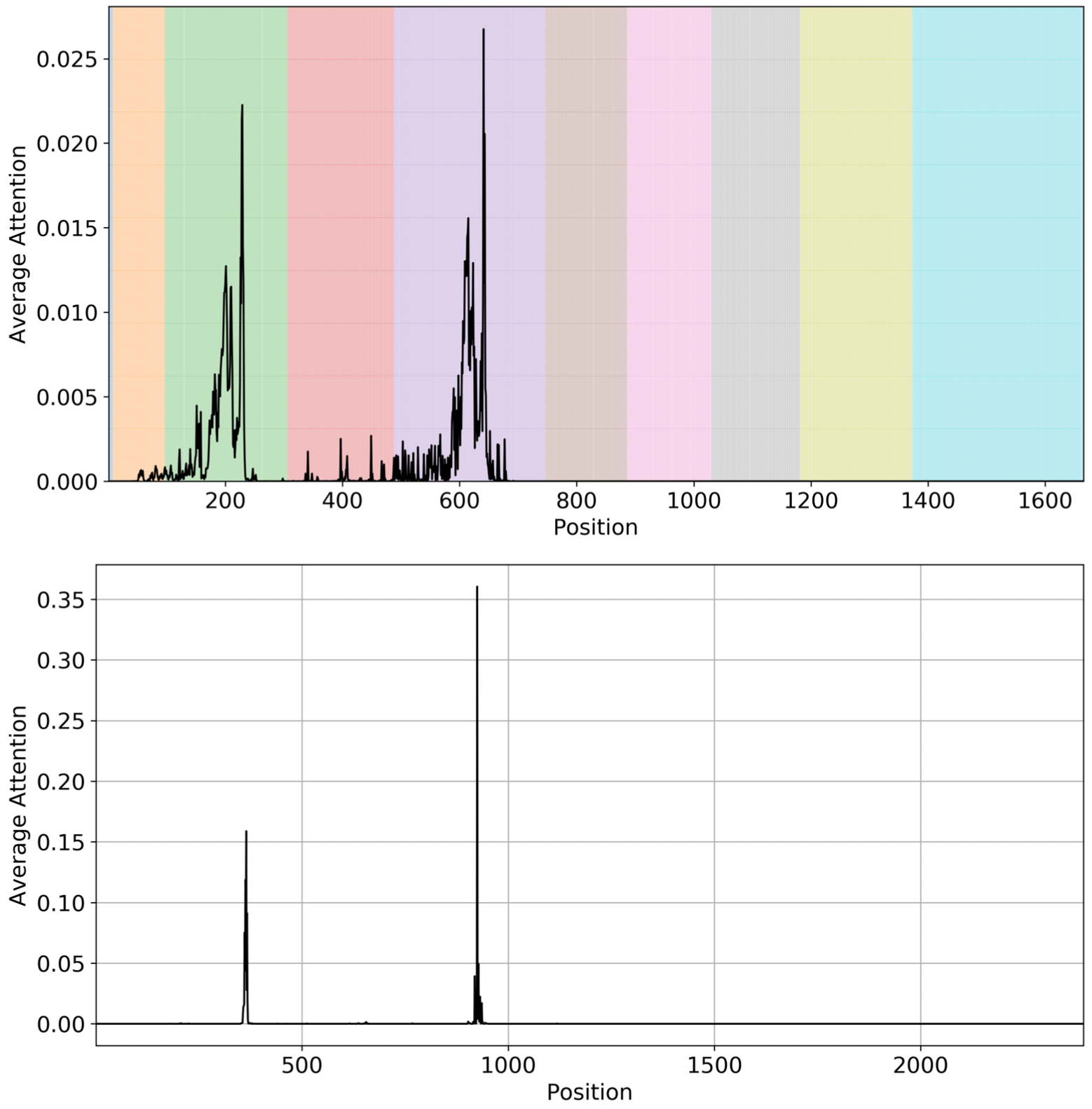

Supplement: S18 Appendix — Top: Average attention weights for all strains of Enterobacter without alignment; Bottom: Attention weights with alignment. This figure shows that our model can implicitly perform alignment to the sequence so that the attention was paid to similar position after alignment. In particular, the model learned to pay attention to important regions. Before alignment, attention are paid to a wider range of positions. However, after sequence alignment, attention weights are concentrated to a smaller range of positions (the maximum averaged attention weight is more than 13 times higher than the weight pre-alignment). This indicates that the model recognizes certain nucleotide configuration and is robust to insertions and deletions which would be accounted for by the sequence alignment. (TIF) [file pcbi.1009345.s018.tif]

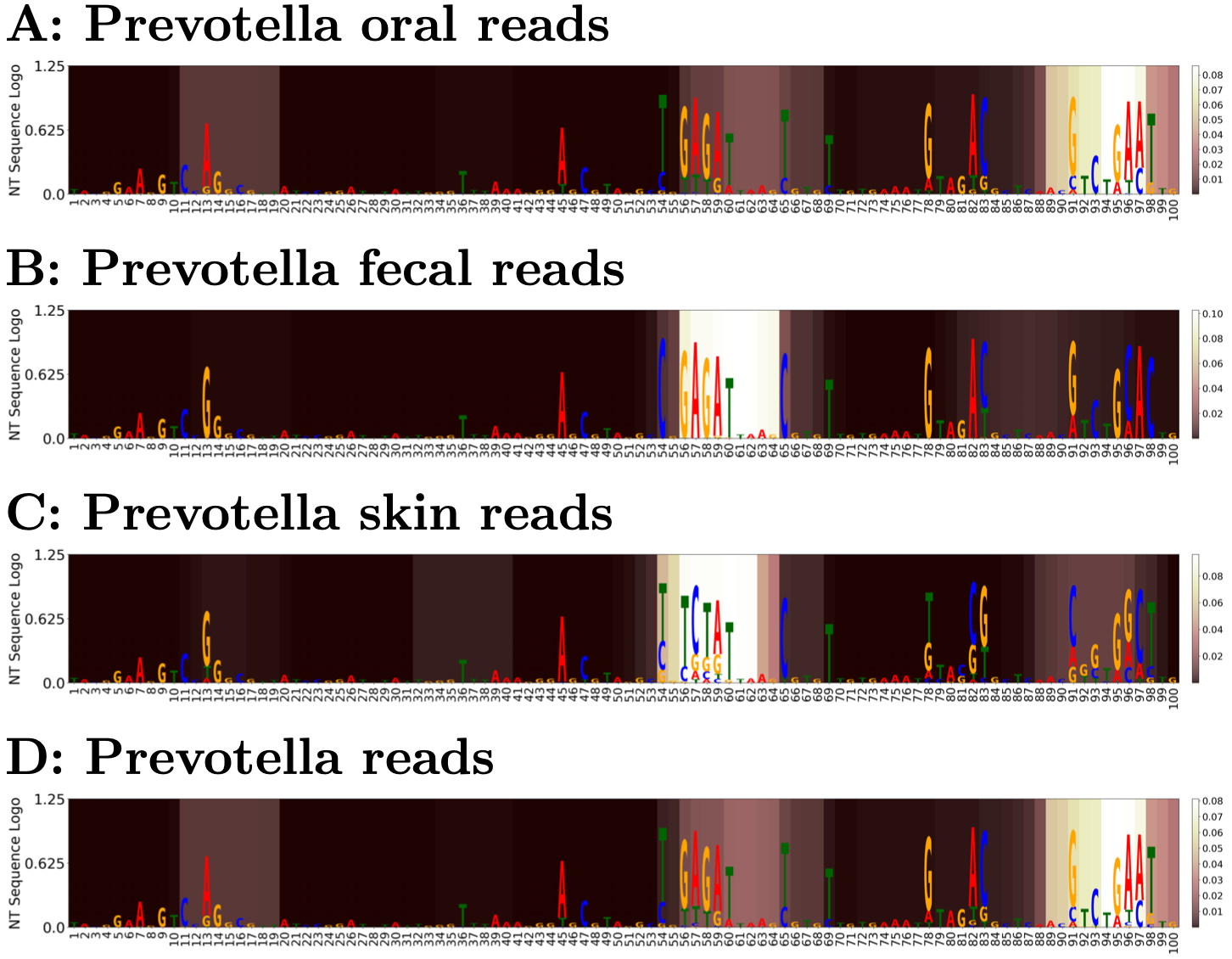

Supplement: S22 Appendix — This figure compares the average Prevotella reads attention and nucleotide frequency entropy in form of nucleotide sequence logo. A: oral reads; B: fecal reads; C: skin reads; D: overall attention. The Prevotella visualization reads are classified to the correct body site source with 68.46% accuracy (the balance accuracy is 66.45%). (TIF) [file pcbi.1009345.s022.tif]

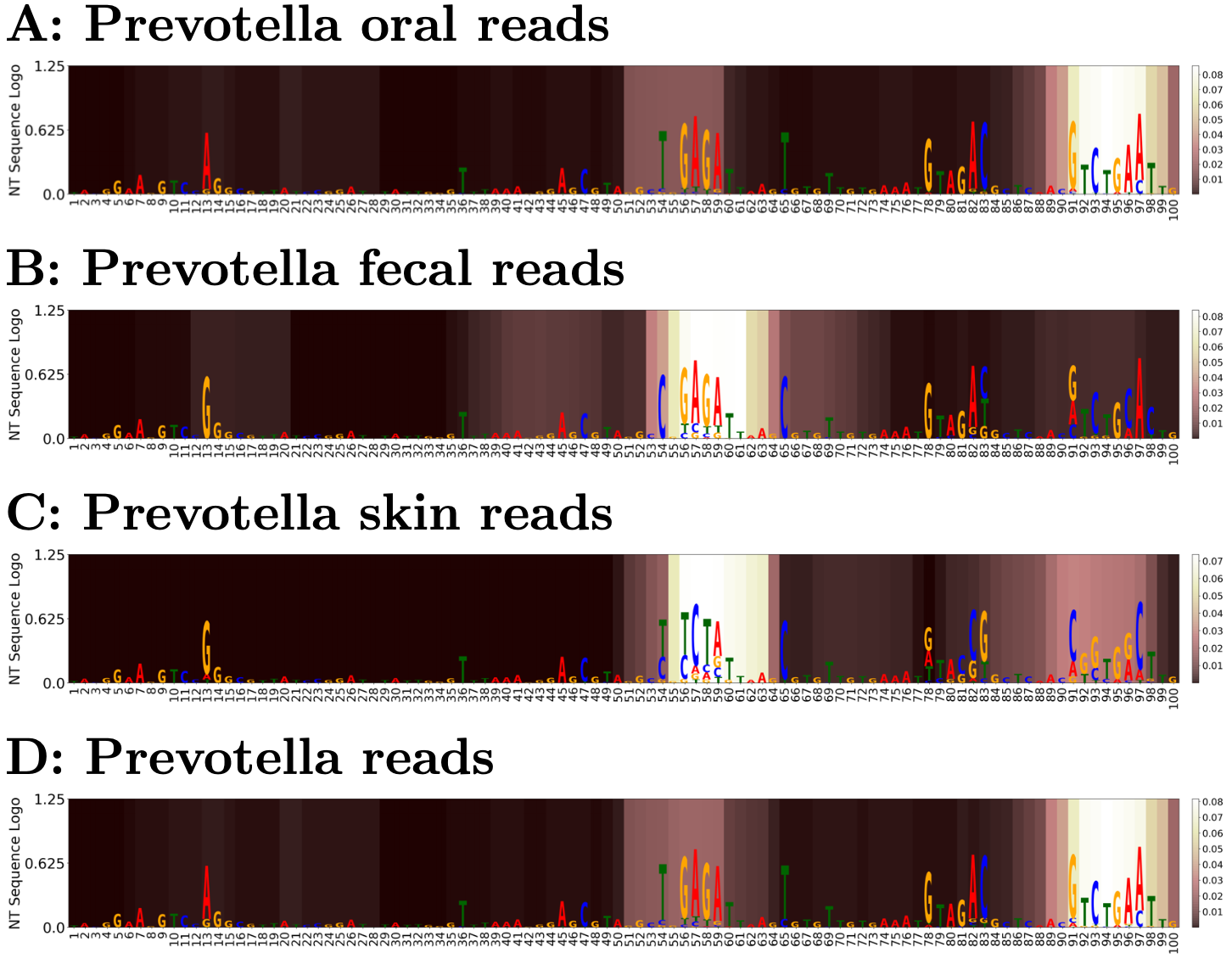

Supplement: S23 Appendix — This figure compares the average Prevotella reads attention and nucleotide frequency entropy in form of nucleotide sequence logo. A: oral reads; B: fecal reads; C: skin reads; D: overall attention. The Prevotella visualization reads are classified to the correct body site source with 91.49% accuracy (the balance accuracy is 67.86%). (TIF) [file pcbi.1009345.s023.tif]

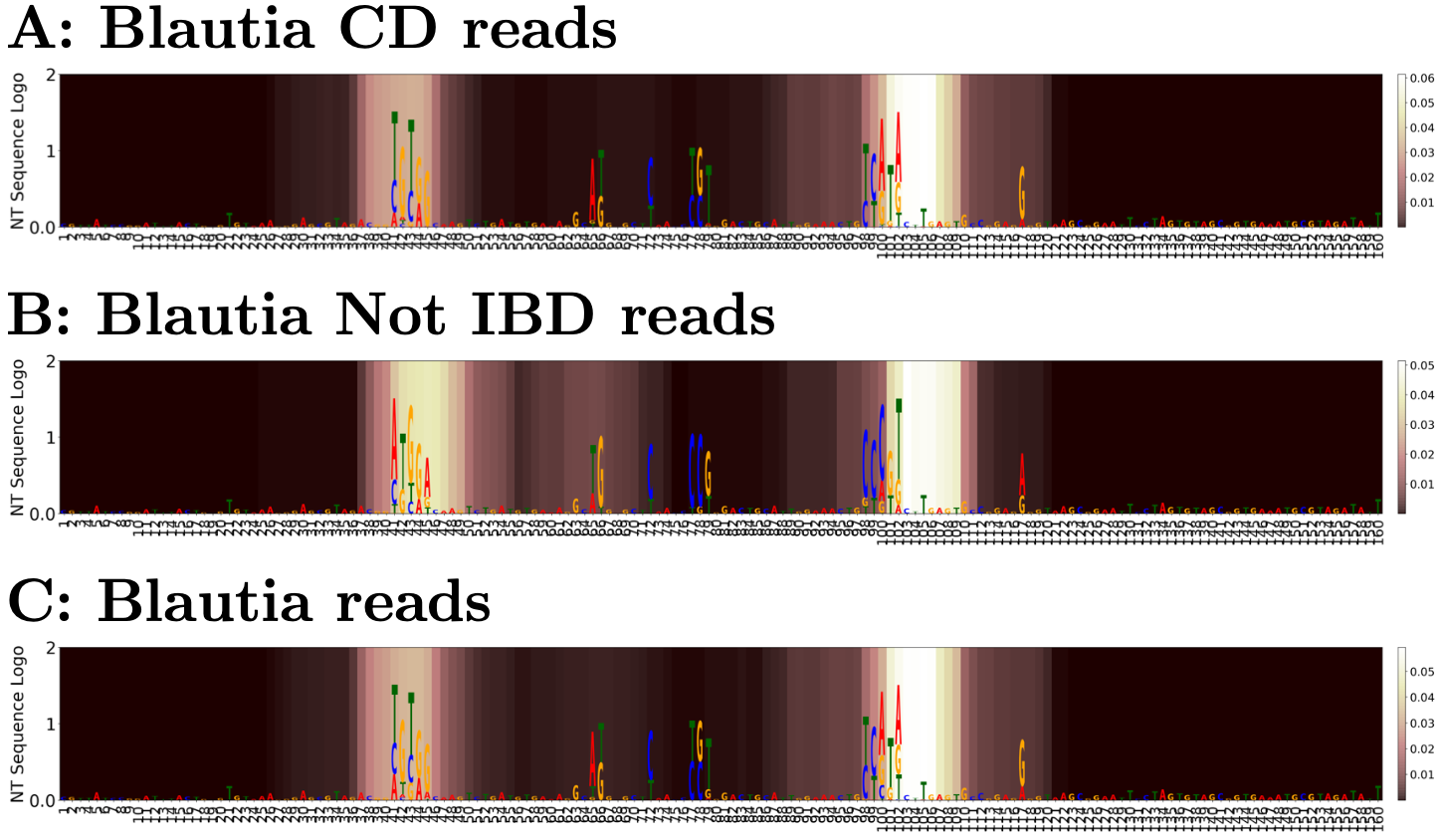

Supplement: S24 Appendix — This figure compares the average Blautia reads attention and nucleotide frequency entropy in form of nucleotide sequence logo. A: “CD” reads; B: “Not IBD” reads; C: overall attention. The Blautia visualization reads are classified to the correct host health status with 6.49% accuracy (the balance accuracy is 22.41% due to high false negative rate on this genus). (TIF) [file pcbi.1009345.s024.tif]

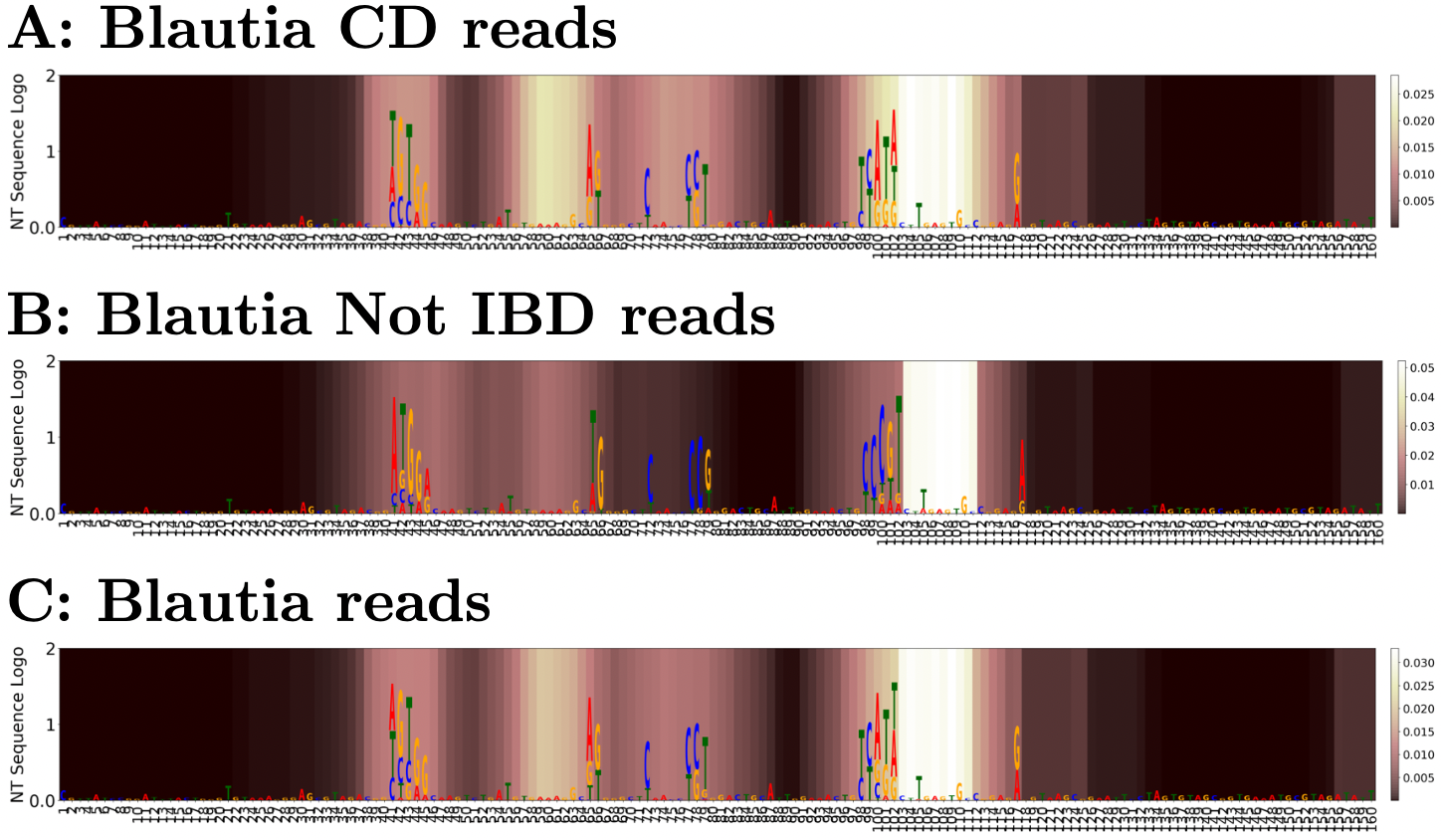

Supplement: S25 Appendix — This figure compares the average Blautia reads attention and nucleotide frequency entropy in form of nucleotide sequence logo. A: “CD” reads; B: “Not IBD” reads; C: overall attention. The Blautia visualization reads are classified to the correct host health status with 75.34% accuracy (the balance accuracy is 48.93%). (TIF) [file pcbi.1009345.s025.tif]
