# Supplementary material for: Learning, visualizing and exploring 16S rRNA structure using an attention-based deep neural network
Source: PLoS Comput Biol. 2021 Sep 22;17(9):e1009345. doi: 10.1371/journal.pcbi.1009345 (PMC8496832; doi:10.1371/journal.pcbi.1009345)
Supplement: S11 Appendix — In this table, we compare the performance of sample phenotype prediction on American Gut Project data as the window size of convolutional layers is varied (i.e., varying the hyperparameter, W, in Fig 1). In this experiment, we evaluated the model with 4 other window sizes, namely, 3, 6, 12 and 15, in addition to our default windows size, 9. We train and evaluate all different models with the 50-sample experimental AGP data set used in Table 2. Similarly, prediction accuracies are averaged and standard deviation is measured over 5 randomly selected data with replacement experiments. The table shows that the sample-level prediction accuracy is generally not sensitive to the window size parameter of the Read2Pheno model. (PDF) [file pcbi.1009345.s011.pdf]

Evaluation of sensitivity of `Read2Pheno` modeling to different window sizes of convolutional layers. In this table, we compare the performance of sample phenotype prediction on American Gut Project data as the window size of convolutional layers is varied (i.e., varying the hyperparameter, W, in Figure 1). In this experiment, we evaluated the model with 4 other window sizes, namely, 3, 6, 12 and 15, in addition to our default windows size, 9. We train and evaluate all different models with the 50-sample experimental AGP data set used in Table 2. Similarly, prediction accuracies are averaged and standard deviation is measured over 5 randomly selected data with replacement experiments. The table shows that the sample-level prediction accuracy is generally not sensitive to the window size parameter of the `Read2Pheno` model.

Method	Window Size				
	3	6	9	12	15
Majority vote	0.727 (± 0.047)	0.731 (± 0.035)	0.730 (± 0.040)	0.736 (± 0.036)	0.736 (± 0.035)
Sample embedding	0.749 (± 0.016)	0.755 (± 0.017)	0.751 (± 0.012)	0.756 (± 0.023)	0.749 (± 0.014)
Pseudo OTU	0.797 (± 0.036)	0.788 (± 0.038)	0.784 (± 0.039)	0.783 (± 0.034)	0.783 (± 0.034)
