# Supplementary material for: Learning, visualizing and exploring 16S rRNA structure using an attention-based deep neural network
Source: PLoS Comput Biol. 2021 Sep 22;17(9):e1009345. doi: 10.1371/journal.pcbi.1009345 (PMC8496832; doi:10.1371/journal.pcbi.1009345)
Supplement: S16 Appendix — To demonstrate the scalability of the Read2Pheno model to longer read lengths, we determined the sample-level classification accuracy of the Read2Pheno model trained on two different read lengths, 160 bp and 250 bp. The analysis shown here is similar to that used to generate Table 3, i.e., the obtained accuracy values are averaged and standard deviation computed over 5 experiments, in which we randomly selected training-testing data splits with replacement. The similarity in trend and values in the table demonstrate that even when trained on reads increased to 250 bp length, Read2Pheno model show stable performance, as well as increased sample-level classification accuracy. As the table showing values for 160 bp and demonstrates, our proposed method can scale to a data set with longer-length reads length data set and can produce better performance with more sequential information from longer read lengths. (PDF) [file pcbi.1009345.s016.pdf]

Gevers data set Testing Performance on different read length. To demonstrate the scalability of the `Read2Pheno` model to longer read lengths, we determined the sample-level classification accuracy of the `Read2Pheno` model trained on two different read lengths, 160 bp and 250 bp. The analysis shown here is similar to that used to generate Table 3, i.e., the obtained accuracy values are averaged and standard deviation computed over 5 experiments, in which we randomly selected training-testing data splits with replacement. The similarity in trend and values in the table demonstrate that even when trained on reads increased to 250 bp length, `Read2Pheno` model show stable performance, as well as increased sample-level classification accuracy. As the table showing values for 160 bp and demonstrates, our proposed method can scale to a data set with longer-length reads length data set and can produce better performance with more sequential information from longer read lengths.

		Training Set Size (samples)		
Category	Method	40	160	400
160 bp model	Majority vote	0.653 ($\pm$ 0.043)	0.690 ($\pm$ 0.023)	0.729 ($\pm$ 0.062)
	Sample embedding	0.650 ($\pm$ 0.016)	0.726 ($\pm$ 0.029)	0.762 ($\pm$ 0.069)
	Pseudo OTU	0.689 ($\pm$ 0.031)	0.779 ($\pm$ 0.014)	0.833 ($\pm$ 0.058)
250 bp model	Majority vote	0.669 ($\pm$ 0.044)	0.711 ($\pm$ 0.023)	0.776 ($\pm$ 0.092)
	Sample embedding	0.694 ($\pm$ 0.034)	0.734 ($\pm$ 0.013)	0.819 ($\pm$ 0.036)
	Pseudo OTU	0.711 ($\pm$ 0.040)	0.800 ($\pm$ 0.015)	0.838 ($\pm$ 0.076)
