# Supplementary material for: Learning, visualizing and exploring 16S rRNA structure using an attention-based deep neural network
Source: PLoS Comput Biol. 2021 Sep 22;17(9):e1009345. doi: 10.1371/journal.pcbi.1009345 (PMC8496832; doi:10.1371/journal.pcbi.1009345)
Supplement: S19 Appendix — The attention weights (top) on a real Pseudomonas aeruginosa sequence provided by [68]. The positions that have an attention weight greater than the mean attention weight across the whole sequence are highlighted on the secondary structure figure (bottom). (PDF) [file pcbi.1009345.s019.pdf]

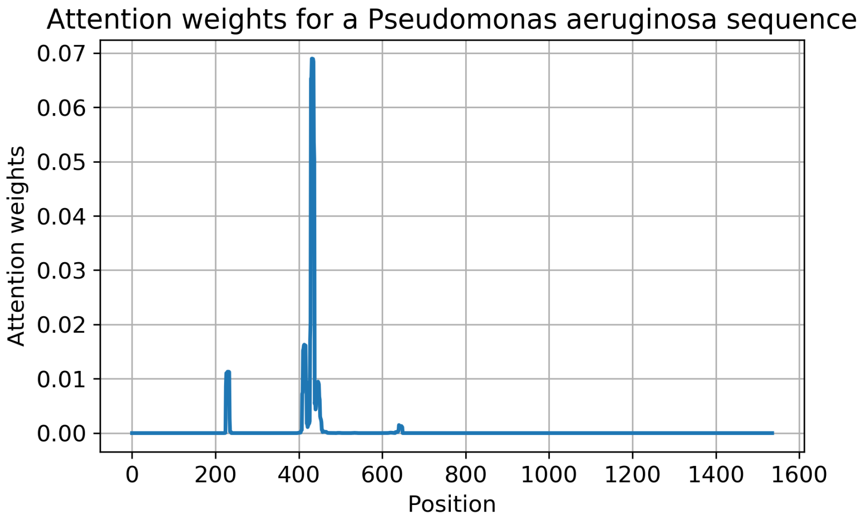
Attention weights for a Pseudomonas aeruginosa sequence
Attention weights
0.07
0.06
0.05
0.04
0.03
0.02
0.01
0.00
Position
0
200
400
600
800
1000
1200
1400
1600

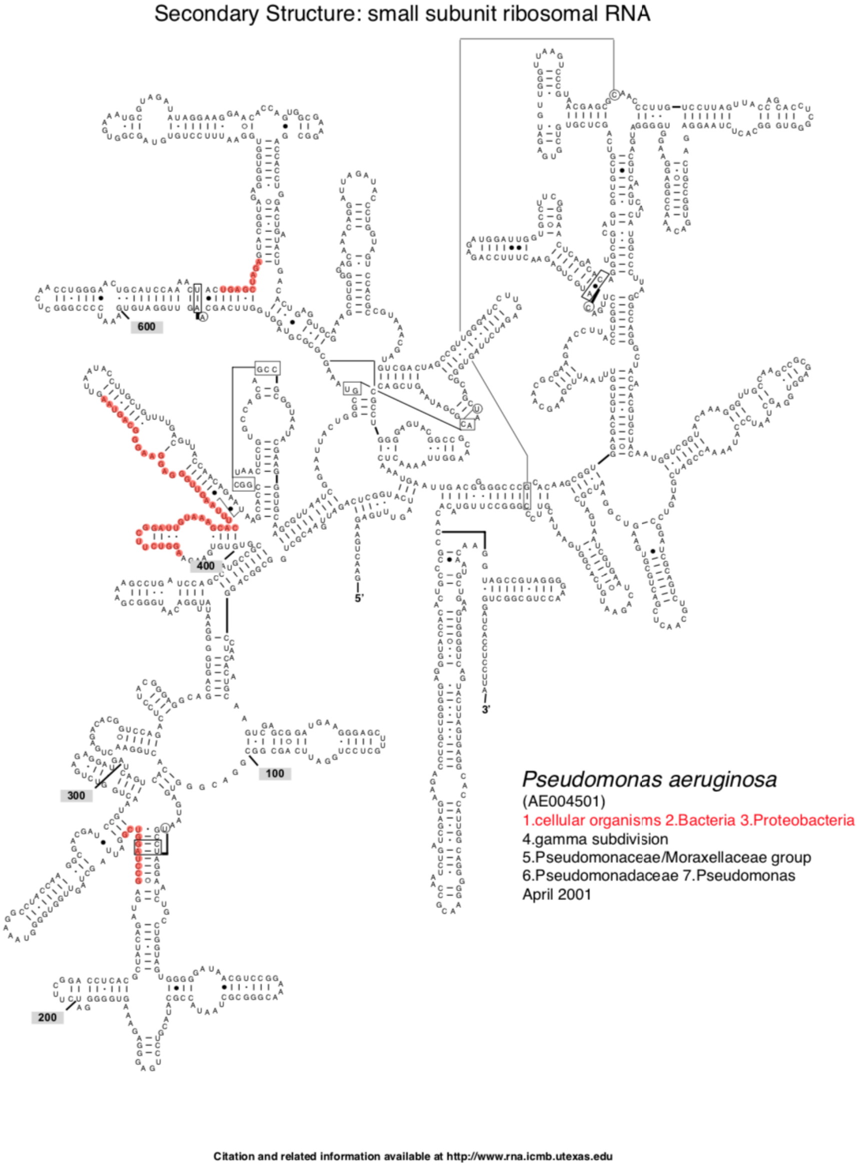
Secondary Structure: small subunit ribosomal RNA
600
400
300
200
100
5'
3'
Pseudomonas aeruginosa
(AE004501)
1.cellular organisms 2.Bacteria 3.Proteobacteria
4.gamma subdivision
5.Pseudomonaceae/Moraxellaceae group
6.Pseudomonadaceae 7.Pseudomonas
April 2001
Citation and related information available at http://www.rna.icmb.utexas.edu
